# Learning-based personalisation of robot behaviour for robot-assisted therapy

**DOI:** 10.3389/frobt.2024.1352152

**Published:** 2024-04-08

**Authors:** Michał Stolarz, Alex Mitrevski, Mohammad Wasil, Paul G. Plöger

**Affiliations:** Autonomous Systems Group, Department of Computer Science, Hochschule Bonn-Rhein-Sieg, Sankt Augustin, Germany

**Keywords:** robot personalisation, robot behaviour model, user modelling, reinforcement learning, assistive robotics

## Abstract

During robot-assisted therapy, a robot typically needs to be partially or fully controlled by therapists, for instance using a Wizard-of-Oz protocol; this makes therapeutic sessions tedious to conduct, as therapists cannot fully focus on the interaction with the person under therapy. In this work, we develop a learning-based behaviour model that can be used to increase the autonomy of a robot’s decision-making process. We investigate reinforcement learning as a model training technique and compare different reward functions that consider a user’s engagement and activity performance. We also analyse various strategies that aim to make the learning process more tractable, namely i) behaviour model training with a learned user model, ii) policy transfer between user groups, and iii) policy learning from expert feedback. We demonstrate that policy transfer can significantly speed up the policy learning process, although the reward function has an important effect on the actions that a robot can choose. Although the main focus of this paper is the personalisation pipeline itself, we further evaluate the learned behaviour models in a small-scale real-world feasibility study in which six users participated in a sequence learning game with an assistive robot. The results of this study seem to suggest that learning from guidance may result in the most adequate policies in terms of increasing the engagement and game performance of users, but a large-scale user study is needed to verify the validity of that observation.

## 1 Introduction

### 1.1 Motivation

One of the objectives of robot-assisted therapy (RAT) (Esteban et al., 2017) is increasing the autonomy of the robot that is used during therapy sessions; this has the purpose of reducing the necessary therapist interactions with the robot (Robins et al., 2017; Rudovic et al., 2017; David et al., 2018; Marinoiu et al., 2018)—such as those required when Wizard-of-Oz (Robins et al., 2005) is used for controlling the robot—while still keeping the therapist in control of the sessions at all times. For instance, in the treatment of children with Autism Spectrum Disorder (ASD), RAT focuses on using a robot to facilitate and guide the learning of concepts that affected individuals require in their everyday lives, such as repeating everyday motions or recognising emotions^1^ In the context of RAT, robot programs are usually developed in such a way that they can be used generically for different individuals; however, individuals may have different reactions to specific stimuli and, depending on their concrete needs, may also benefit from therapy sessions focusing on specific aspects. This means that a generic RAT approach may not be optimal for effective treatment of individuals; instead, the robot should be able to adapt its behaviour to the needs of each individual and therapy session (Esteban et al., 2017; Rudovic et al., 2018; Scassellati et al., 2018).

This type of adaptation, also referred to as *personalisation*, requires a robot to modify its behaviour to each individual user or to groups of similar users. A personalised behaviour model can be learned by involving a user in the learning loop, which is referred to as interactive machine learning (Senft et al., 2019). There are two primary types of interactive machine learning in the context of personalisation Tsiakas et al. (2016), namely *learning from user feedback* and *guidance-based learning*, where the former relies on direct or indirect user feedback, while the latter incorporates feedback from an external observer, for instance a therapist. Learning from user feedback can be difficult to perform efficiently because the robot needs to perform exploration to find an appropriate behaviour policy, while guidance-based learning avoids incorrect actions being performed by the robot during the learning process, but may require a supervisor to be involved for prolonged periods of time for a sufficiently good behaviour policy to be learned. One way in which the amount of involvement of a user or a supervisor can be reduced is by incorporating a user model (Rossi et al., 2017) in the policy learning process, based on which users are represented by particular parameters, such as their engagement.

**FIGURE 1:**
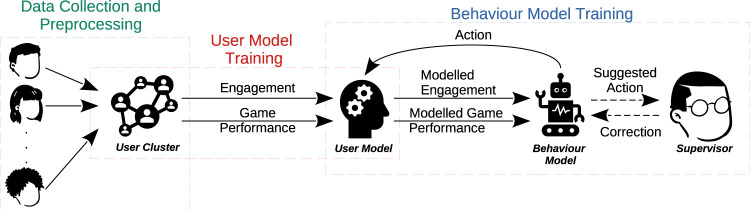
Overview of the proposed interaction and behaviour model learning process. We train user models with data collected during various interaction sessions with a robot. The models are trained to estimate the users’ engagement and performance in a given activity, such that they encode aggregated data for similar users. The user models are used to simulate user interactions during behaviour model training; optionally, expert feedback is also included during the behaviour model learning process.

In this work, we build upon Stolarz et al. (2022a) and present a personalised behaviour model^2^ that a) personalises the difficulty of activities to an individual’s skill level, b) aims to prevent users from getting disengaged by giving appropriate feedback, and c) learns with a small number of interactions with a user. An overview of the developed solution is presented in Figure 1. The developed model is learning-based, using the observed user’s engagement score and activity performance as a learning signal. Our model is particularly based on the concept of learning from feedback and guidance, such that it incorporates learned user models that estimate a user’s engagement and expected performance in an activity. We train the behaviour model with user models learned from real interaction data collected from multiple users, which are split into clusters (data collection and preprocessing phase) and a dedicated user model is learned for each cluster (user model training phase); both the engagement and the performance of users are represented as Gaussian processes. Based on these models, we learn a policy that a robot uses for selecting the difficulty level of an activity and the type of provided feedback to the user (behaviour model training phase). We compare different rewards for the policy learning algorithm and investigate a policy pretraining method for accelerating the policy convergence speed. We also investigate an optional approach, which is learning from guidance, where the supervisor corrects actions before they are executed by the robot. To evaluate the feasibility of the proposed method, we present an experiment with QTrobot (Costa et al., 2017) in which six adult participants were playing an emotion sequence memorisation game. We plan to perform a larger user evaluation as well as experiments with children with autism in subsequent studies.

The contributions of this work include:

• Main contributions:
  • A personalisation pipeline that enables both learning from feedback and learning from guidance to be used for behaviour model learning, either independently or in combination.
  • A learned user model incorporating engagement that is estimated based on visual features; this is in contrast to the related work, where visual features have only been used in rule-based models.
• Further contributions:
  • A comparison of learning from feedback and learning from guidance for training a behaviour model in the context of robot-assisted therapy, where the learned user model is used during learning from feedback, and an investigation of policy transfer for improving the speed of learning from feedback.
  • A small-scale feasibility study with an assistive robot which suggests the potential benefit of personalising behaviour models to individual users; user studies have clearly been performed before, including considerably larger ones, but our study focuses on a comparison of policies acquired using learning from feedback and learning from guidance.

### 1.2 Related work

We analysed various personalisation approaches in terms of the usability for RAT, particularly for children with ASD. Considering only aspects in which the robot control can be adjusted, these can generally be categorised into four personalisation dimensions (Tsiakas et al., 2018b): social behaviour personalisation, game difficulty personalisation, affection personalisation, and personalisation of user preferences (e.g., proxemics). It is particularly desirable that a robot is able to personalise its behaviour such that it improves a user’s performance in therapeutic activities (with or without the help of a supervisor); this can be done by adjusting the difficulty of the activities to each individual user. Additionally, the robot should react properly when the interaction with a user does not go as planned, which means that it should prevent the user from getting bored, disengaged or demotivated, for instance by providing reengaging and motivating feedback. These types of personalisation are referred to as game difficulty and social behaviour personalisation, respectively. In this section, we present literature addressing these two dimensions and discuss its limitations that our work aims to address^3^.

#### 1.2.1 Social behaviour personalisation

Social behaviour personalisation refers to how a robot adapts its gestures, facial expressions, and language content (e.g., type of feedback) to a user. The aim of this personalisation technique is to maintain user involvement in the interaction. One solution for this is the RAT system in Esteban et al. (2017); Cao et al. (2019), where the robot produces actions according to therapeutic scripts defined by a supervisor and, when the interaction does not go as planned, the robot tries to seek appropriate actions on its own (Cao et al., 2019); however, before executing any action, the robot requests a supervisor for feedback about its suitability and includes the feedback into its behavioural model for learning, which makes it similar to the learning from guidance concept. This approach has been successfully deployed and evaluated in real-world scenarios (Cao et al., 2019), but it was not personalised, as the learning procedure was performed on data from all study participants.

One proposed model for decision-making is a feed-forward network (Senft et al., 2015b), which has good generalisation abilities and can be personalised to a specific person (Senft et al., 2015a), but has to be retrained every time supervisor feedback is obtained. This may make this solution inappropriate for real-time interactions in case of long-time scenarios (Senft, 2018), as the learning time increases with the amount of collected data. Another proposed solution is based on reinforcement learning with the Q-learning algorithm (Watkins and Dayan, 1992; Senft et al., 2017); however, in Q-learning, a considerable amount of data is needed to obtain an optimal policy, which means that a significant number of interactions with the user is required. In Senft et al. (2015b), Senft et al. (2017), the behaviour models were evaluated only with people acting as supervisors and not as users interacting with the robot. To guarantee fast convergence of the Q-learning algorithm, the problem has to be decomposed so that the Q-value table stays relatively small (Hemminahaus and Kopp, 2017). To reduce the memory requirements and make the learning algorithm converge faster, the MAXQ hierarchical reinforcement learning algorithm (Dietterich, 2000) is used in Chan and Nejat (2012), where a robot providing personalised assistive behaviours for a memory game is developed. This work presents a strongly task-specific solution, however, which would require significant changes to be adapted to another use case. The deficit of high memory requirements was also faced in Senft et al. (2019), Winkle et al. (2020), where applying nearest neighbours allowed to obtain a reasonable training time. This solution is strongly dependent on the type of the performed activity, as it requires all activity states to be defined in an activity-specific vector space in which distances for the nearest neighbour algorithm can be calculated.

Most of the aforementioned approaches are based on learning from guidance (Senft et al., 2015b; Esteban et al., 2017; Senft et al., 2017; Senft et al., 2019), which is particularly advantageous for systems where robot mistakes imply ethical concerns. This approach is, however, very dependent on the supervisor, which can have a negative impact on the learned policy, especially when the supervisor makes incorrect decisions. The effects of supervisor mistakes can be alleviated if the learning signal is obtained directly from the user. This is done by applying learning from feedback, where the robot has to find appropriate actions on its own. In Chan and Nejat (2012), Leyzberg et al. (2014), Hemminahaus and Kopp (2017), Velentzas et al. (2018), approaches for personalising a robot’s behaviour in real-life scenario based exclusively on learning from feedback are described. In particular, in Leyzberg et al. (2014), the robot was deployed in the role of a tutor that is giving lessons to a user playing a puzzle game; however, this approach is limited to providing users only with lessons that complement their missing knowledge and is not able to react when the interactions do not go as planned, for instance when the user becomes disengaged in the activity. In Velentzas et al. (2018), one of the robot’s tasks was to learn how to execute a pointing action with different levels of expressivity in order to increase a child’s engagement. The authors’ algorithm is designed for non-stationary problems (by switching adequately between exploration and exploitation), and can choose appropriate action (using Q-learning) as well as find suitable action parameters (using actor-critic RL (Van Hasselt and Wiering, 2007)). This learning from feedback solution is not shown to provide more elaborate social interaction (e.g., the robot is not able to provide verbal feedback) and is only tested in simulation. The concept of combining learning from guidance and feedback is also discussed in Tsiakas et al. (2016); however, the presented system is not evaluated with real users and does not perform real-time user engagement estimation.

#### 1.2.2 Activity difficulty personalisation

The methods discussed above are adaptive in terms of the reactions to a user’s behaviour; however, in RAT, it is also important to autonomously adapt the difficulty of activities during interactions in order to match each individual’s skill level. Systems that can provide this type of adaptation are primarily based on learning from feedback. A personalisation concept based on adapting the progression of a lesson to a user’s performance is covered in Jain et al. (2020), Clabaugh et al. (2019), Baxter et al. (2017), Scassellati et al. (2018). Baxter et al. (2017) deploy a rule-based adaptation algorithm, for instance based on a comparison of the number of successfully completed tasks to a predefined threshold; this approach does not allow for learning, as manually written rules are used for choosing an appropriate difficulty level. In Jain et al. (2020); Clabaugh et al. (2019); Scassellati et al. (2018), learning is used in order to personalise the feedback and instruction difficulty levels during various games, but engagement is not taken into account in the game personalisation model.

Another personalisation method based on learning from feedback, which our work builds upon, is presented in Tsiakas et al. (2018a). Here, Q-learning is used so that a robot can adapt the difficulty of a game and provide user feedback; furthermore, this work proposes the use of learned user models for behaviour model training and investigates different methods of updating the Q-table to increase the policy convergence speed. In Tsiakas et al. (2016), it is additionally shown that the number of iterations required for a policy to converge can be reduced by transferring learned policies between users. The techniques in Tsiakas et al. (2016) and Tsiakas et al. (2018a) are, however, not evaluated with real users.

#### 1.2.3 Addressed challenges

The above discussion of work on personalisation for RAT illustrates various challenges that reduce the applicability of learning-based personalisation strategies in real-life interventions. This includes i) the maintenance of an adequately large state and action space, enabling the robot to personalise the game difficulty and its reactions to a specific user, ii) fast convergence to an optimal policy without the need for a significant number of interactions with a user, or finding a sufficiently good policy for effective practical interaction, and iii) evaluation during interactions with real users.

In this paper, we present a learning-based behaviour model for RAT that allows a robot to autonomously personalise the difficulty of an activity based on a user’s individual set of skills, but also enables the robot to react to an individual’s perceived disengagement by providing appropriate encouraging or challenging feedback during the activity. For this, we build upon the Q-learning method proposed in Tsiakas et al. (2018a), such that we investigate behaviour model pretraining based on policy transfer, similar to Tsiakas et al. (2016). We additionally apply learning from guidance, based on the control sharing method introduced in Knox and Stone (2012), to enable the behaviour model learning process to converge fast, while also enabling supervisors to direct the robot’s behaviour based on their preferences. Our method is evaluated in a small user study that demonstrates the feasibility of learning-based personalisation, particularly when the model learning process is guided by a supervisor.

A summary of the main differences of our approach with respect to the related work is provided in Table 1.

**TABLE 1:** Comparison of our method with respect to the related work on personalisation. (*✓*) denotes that the approach was only evaluated with people acting as supervisors and not as users interacting with the robot.

References	User model	Engagement features	Policy transfer	Guidance used	User study	End users involved
Tsiakas et al. (2016)	Rule-based	✗	*✓*	*✓*	✗	✗
Tsiakas et al. (2018a)	Neural network & Support Vector Regression	EEG data	✗	✗	✗	✗
Clabaugh et al. (2019); Hemminahaus and Kopp (2017); Leyzberg et al. (2014); Baxter et al. (2017); Scassellati et al. (2018)	✗	✗	✗	✗	*✓*	*✓*
Jain et al. (2020)	✗	Visual, audio and game performance	✗	✗	*✓*	*✓*
Senft et al. (2015a)	Rule-based	Calculated from model	✗	*✓*	✗	✗
Senft et al. (2015b)	Rule-based	Visual features	✗	*✓*	(*✓*)	✗
Senft et al. (2017)	✗	✗	✗	*✓*	(*✓*)	✗
Senft et al. (2019)	✗	✗	✗	*✓*	*✓*	*✓*
Winkle et al. (2020)	✗	Heart rate, motivation/effort, visual features	✗	*✓*	*✓*	*✓*
Chan and Nejat (2012)	Bi-gram	✗	✗	✗	*✓*	✗
Velentzas et al. (2018)	Rule based, probabilistic	Visual features	*✓*	✗	✗	✗
Stolarz et al. (2022a)	Gaussian process	Visual features	*✓*	✗	✗	✗
**Our work**	**Gaussian process**	**Visual features**	*✓*	*✓*	*✓*	✗

## 2 Materials and methods

The objective of this work is to develop a personalisation strategy for RAT, with a particular focus on adapting the behaviour of a robot in terms of controlling the activity difficulty and providing appropriate user feedback. For this purpose, we present a method for learning a robot behaviour policy that incorporates a learned user model in the policy learning loop. In this section, we first introduce a robot-assisted game, which is the use case for our behaviour model. We then introduce a formulation of the behaviour personalisation problem, describe a classifier for estimating the engagement of a user, and elaborate on the design of user models that estimate a user’s engagement and expected performance in an activity. Next, we describe a basic version of the behaviour model which is based on the concept of learning from feedback, and then introduce two methods for improving the policy convergence speed, namely i) policy transfer and ii) learning from guidance. An overview of the notation introduced in this section and used throughout the paper is given in Table 2.

**TABLE 2:** Overview of the notation introduced in this section.

Symbol	Meaning	Symbol	Meaning
M	User model	B	Behaviour model
F^p^	Performance prediction component of a user model	**s** ^P^	Activity state vector; input to F^p^
*F* ^*e*^	Engagement prediction component of a user model	**s** ^*e*^	Engagement state vector; input to *F* ^*e*^
E	Engagement estimator	*n*_*E*_	Number of engagement estimates within a given time frame
**u**	User vector representing success probabilities and engagement values for different difficulty levels	*p*_*j*,*m*_	Probability that user *j* correctly solves a sequence of length *m*
*L*	Sequence length	*E*	Estimated engagement
*F*	Feedback type	*PS*	Raw activity score
*t*	Current time step	**s**_*t*_	State of B at time *t*
*R*_*a*_(**s**_*t*_)	Immediate reward received after applying action *a* in state ***s**_t_*	*RE*	Activity reward
*F*_*r*_	Reward function	$T_{s_{t}}$	Temperature parameter for state **s**_*t*_
*O*	Outcome of solving a sequence	*Q*	Robot policy Q-table
*n*	Number of users	$\hat{H}$	Supervisor policy
*a*_*t*_	Action selected by the robot’s policy	*a*_*c*_	Action selected by the supervisor’s policy
*δ*	Combination parameter for control sharing	*β*	Engagement weight for *F*_*r*_
*C*	User cluster	Ω	Sequence of words
*c*	Number of user clusters	*ω*	Number of sequences
*k*	Cluster ID	*l*	Maximum activity difficulty level

### 2.1 Robot-assisted game use case

To ground the personalised behaviour model to a concrete task, we use a game whose objective is to evaluate the ability of users to memorise and repeat sequences of spoken emotions; a game of this type has also been used in Scassellati et al. (2018). Our game is designed based on Tsiakas et al. (2018a), such that each user session consists of *ω* = 10 sequences to memorise. Each sequence Ω_*j*_, 1 ≤ *j* ≤ *ω* consists of words that are randomly sampled from a pool of four emotions, namely {happy, disgusted, sad, angry}; a sequence can have a length |Ω_*j*_| of 3, 5 or 7 emotions, with respective difficulty levels *L*_*j*_ ∈ {1, 2, 3}. The lengths were chosen to provide sequences commonly considered easy, just right (but not easy), and difficult; we investigate whether this hypothesis holds in our experimental analysis. During the game, the robot says each Ω_*j*_ out loud and the user has to reproduce the sequence by selecting images corresponding to the emotions on a tablet. To reproduce a sequence correctly, the user has to choose the correct image for every emotion in Ω_*j*_ in the right order. During the game, the robot should choose sequence lengths |Ω_*j*_| that are appropriate for the user and provide feedback *F*_*j*_ so that the user remains engaged in the interaction; thus, we want the selection of robot actions to be based on the user’s game performance and engagement level.

We use QTrobot as a robotic assistant in this work, which is a robot developed for tablet-based interactive games. The robot has an Intel RealSense D435 depth camera, a Raspberry Pi, and also includes an Intel NUC PC for more demanding computations. QTrobot is integrated with two tablets—one for the educator and one for the user; this allows educators to control the robot or start appropriate games during a session, while the user tablet is only supposed to execute the games chosen by the educator.

### 2.2 Formulation of behaviour personalisation

The purpose of using a user model is to reduce the amount of user or educator interactions that are needed for learning a behaviour policy. We utilise a user model that estimates the engagement and expected performance of a group of similar users in a given activity, assuming that both performance and engagement are represented by numerical values.

Definition 1. A user model M is a tuple $M=\left(F^{p},F^{e}\right)$ , where *F* ^*p*^ is a performance prediction component and *F* ^*e*^ is an engagement estimation component.

A model M is learned from user data collected during real interactions, where the estimated engagement and activity performance are recorded. These data are then clustered in order to identify groups *C*_*k*_, 1 ≤ *k* ≤ *c* of similar users, namely users that have similar performance and engagement during an activity, where *c* is the number of user groups. For each *C*_*k*_, both user model components are learned from the data as Gaussian processes (GPs) (Rasmussen and Williams, 2006), which have the desirable property of encoding prediction uncertainty. The learned user model is then incorporated into a policy learning loop, such that the policy *π* is learned using Q-learning on discrete state and action spaces. In the rest of this section, we describe the engagement estimation, the model M , and the policy learning in more detail.

### 2.3 Engagement estimation

Behaviour models need external information to adjust the robot reactions (e.g., feedback for the user). The user’s affective state can be used for that purpose and is usually modelled by three factors: valence, arousal and dominance (Cao et al., 2018). Valence, which describes the positiveness of emotion, is useful along with engagement (Gordon et al., 2016), such that there are various methods of estimating them (Rudovic et al., 2018; Jain et al., 2020); however, in various applications, using the affective state directly may result in a suboptimal behaviour model, such as in the case of children with autism, who usually have difficulties recognising and expressing emotions (Rudovic et al., 2017). For this reason, engagement is the feature that is often used for the development of behaviour models (Senft et al., 2015a; Senft et al., 2015b; Tsiakas et al., 2018a). One way to measure engagement is with the use of an EEG headset (Tsiakas et al., 2018a), but an external engagement observer might be more convenient and simpler for users, as they may otherwise be distracted by the additional equipment; this may be a problem during therapy for children with ASD (Javed et al., 2019), but can also be too cumbersome for everyday deployment.

To estimate the engagement of a user during an activity, we use a binary classifier $E:R^{32}\to \left\{-1,1\right\}$ based on Jain et al. (2020); here, 1 denotes engagement, namely that the participant is actively involved in the interaction and pays attention to the robot, while −1 denotes disengagement, namely that the participant is not focused on the robot (this includes cases such as putting the head down on the table, turning the head away from the robot, or standing up and walking away). For the classifier, we use 32 features—head pose (6 features), facial action units (18 features), and gaze position and angle (8 features); these are extracted using the OpenFace library (Baltrusaitis et al., 2018). To collect training data for the classifier, we asked participants to act out the aforementioned engagement and disengagement criteria; this has the potential disadvantage that the participants’ behaviour may not be completely natural during the interaction, but simplifies the data labelling effort since the interactions are appropriately segmented during data collection. Using the training data, we performed an evaluation procedure similar to Jain et al. (2020) to select a suitable classifier. We particularly performed leave-one-out cross validation and compared multiple classifier types. Based on this evaluation, we use an XGBoost^4^ classifier in this study, which has a validation accuracy of about 85%^5^.

Here, it is important to mention that our system returns an engagement score several times per second; however, in order to prevent noise from affecting the state estimate, we make an assumption that a person’s affective state would not significantly change within one second^6^. Thus, instead of using the raw estimates directly, we use an *expected engagement* value that is calculated for every second of the interaction as

$$\text{E}\left[E\right]=\underset{i}{\sum }e_{i}P\left(E=e_{i}\right)$$ (1)

Here, *e*_*i*_ stands for one of the possible engagement scores, so *i* ∈ {0, 1} and *e*_*i*_ ∈ {−1, 1}, such that

$$P\left(E=e_{i}\right)=\frac{n_{e_{i}}}{n_{E}}$$ (2)

where $n_{e_{i}}$ is the number of times (within the considered second) when the engagement score was *e*_*i*_ and *n*_*E*_ is the total number of measurements, namely $n_{E}=\sum_{i}n_{e_{i}}$ . It should be noted that we use an expected engagement calculation instead of a simple majority vote so that we have a continuous estimate that more accurately reflects the real state of the user, which may sometimes be ambiguous. In addition, having a continuous engagement value is beneficial for integrating the engagement into a reward function such as the one used in section 3.1.5.

### 2.4 User model

Evaluating the behaviour model on real users is a time-consuming and expensive process; thus, user simulations are often used for testing purposes (Senft et al., 2015a; Tsiakas et al., 2016; Tsiakas et al., 2018a). The user model presented in Senft et al. (2015a) is a rule-based child interaction model, which assumes that the child state is defined by three variables: *E* (engagement), *M* (motivation) and *P* (performance). Here, the motivation level is a variable related to the speed of solving tasks (Senft et al., 2015b), while performance can be understood as a measure of the user’s success during an activity. This model is, however, manually designed and only motivated by real-life interaction data; thus, it does not entirely capture real interactions between a robot and a user and may be unsuitable to represent the individual characteristics of each specific user. More realistic is the rule-based model presented in Tsiakas et al. (2016), which is given in the form of a table. This model’s output is binary and indicates the success or failure of a user while solving a task with a certain difficulty level and duration time; however, the model only captures the changes in the game performance of the user, while the user’s engagement is not considered. A more suitable approach is presented in Tsiakas et al. (2018a), where user models were fitted to data collected during user evaluations. The experiments included a sequence learning task, where each participant had to recreate a sequence consisting of three different letters. A neural network is used to create a user’s game performance model and Support Vector Regression to create an engagement model for the user.

For creating a user model, we followed Tsiakas et al. (2018a); however, we are using a GP, as we found out that it was able to fit the data much better than other regressors^7^. Given a dataset *X* for *n* users, where the activity scores, solved difficulty levels, estimated engagement values, and robot feedback types were recorded throughout an activity, we i) represent each user as a vector, ii) standardise the vectors and perform dimensionality reduction to 2D space, iii) cluster the users into *c* groups, and iv) train *c* models $M_{k}$ (one for each user cluster) to predict activity performance and engagement scores for unseen activity states.

We represent each user *U*_*j*_, 1 ≤ *j* ≤ *n* by a vector **u**_*j*_

$$u_{j}=\left(p_{j,1:l},e_{j,1:l}\right)$$ (3)

where *p*_*j*,1:*l*_ are the success probabilities of solving each of the sequence difficulty levels *L*_*i*_, 1 ≤ *i* ≤ *l* and *e*_*j*,1:*l*_ is the user’s mean engagement score for the respective difficulty levels^8^. We then project the vectors **u**_*j*_ for all *n* users onto a *z*-dimensional space using principal component analysis (PCA)^9^ and apply K-means clustering to group the projected vectors into *c* clusters. Given the assignment of users *U*_*j*_ to a cluster *C*_*k*_, we create a performance model $F_{k}^{p}$ and an engagement model $F_{k}^{e}$ ; these comprise the user model $M_{k}$ for cluster *C*_*k*_. Both $F_{k}^{p}$ and $F_{k}^{e}$ are GPs used for regression to unobserved states, namely

$$F_{k}^{p}\left(s^{p}\right)=GP\left(\mu \left(s^{p}\right),\kappa \left(s^{p},s^{p\prime }\right)\right)$$ (4)

$$F_{k}^{e}\left(s^{e}\right)=GP\left(\mu \left(s^{e}\right),\kappa \left(s^{e},s^{e\prime }\right)\right)$$ (5)

where *μ* is the mean and *κ* the covariance of the inputs. It is essential to mention that the user model is trained on data collected during the sequence learning game; on the other hand, the engagement estimator is trained on a dedicated dataset in which participants were acting out criteria that were manually determined to represent engaged or disengaged behaviour.

#### 2.4.1 Performance model

$F_{k}^{p}$ predicts how likely the users in *C*_*k*_ are to succeed in a given activity state **s** ^*p*^, namely $F_{k}^{p}\left(s^{p}\right)\mapsto \left[0,1\right]$ , where each **s** ^*p*^ = (*L*, *F*, *PS*). Here, *L* ∈ {1, …, *l*} is the current difficulty level, *F* ∈ {0, 1, 2} is the given robot feedback (no feedback, encouraging feedback, or challenging feedback, respectively), and *PS* ∈ {−*l*, − *l* + 1, …, 0, …, *l* − 1, *l*} is the activity score achieved by the user in the last sequence^10^.

#### 2.4.2 Engagement model

$F_{k}^{e}$ estimates the expected engagement value for the users in *C*_*k*_, namely $F_{k}^{e}\left(s^{e}\right)\mapsto \left[-1,1\right]$ , where **s** ^*e*^ = (*L*, *F*, *PS*, *O*). Here, *O* ∈ {1, − 1} stands for the outcome (correct or wrong) of solving the current sequence.

### 2.5 Robot behaviour model

Our behaviour model for robot decision making is represented as a discrete Markov Decision Process $B=\left(S,A,P_{a},R_{a},\gamma \right)$ , where each state **s** ∈ *S* is defined as **s** = (*L*, *F*, *PS*) and the action space *A* consists of actions *a*_*i*_, 1 ≤ *i* ≤ *l* + 2. Here, actions *a*_1:*l*_ are used to set a difficulty level *L* for the next sequence, while actions *a*_*l*+1_, *a*_*l*+2_ say either encouraging (*a*_*l*+1_) or challenging feedback (*a*_*l*+2_) and repeat the same *L* for the next sequence^11^. The robot in state **s**_*t*_ moves to a state **s**_*t*+1_ after performing action *a* with probability *P*_*a*_(**s**_*t*+1_|**s**_*t*_, *a*) and receives an immediate reward *R*_*a*_(**s**_*t*_), which can be based on i) the activity result *RE*_*b*_ ∈ {−1, 1, …, *l*}, where −1 is given for a wrong answer and 1, …, *l* for a correctly solved sequence of level *L*, and ii) the mean engagement score *E* ∈ [−1, 1] calculated after the user solves a given sequence^12^. Finally, *γ* is a discount factor.

We perform model learning with the tabular Q-learning RL technique, which is summarised in Algorithm 1. Here, *Q*(**s**_*t*_, *a*_*t*_) is the value of a given entry in the Q-value table, **s**_*t*_ and **s**_*t*+1_ are the states before and after the execution of *a*_*t*_, respectively, and *R*_*a*_(**s**_*t*_) is the immediate reward after applying *a*_*t*_ in **s**_*t*_. *R*_*a*_(**s**_*t*_) is calculated by a function $F_{r}:\left(R,R\right)\to R$ , such that we experiment with different functions in the evaluation. The action *a*_*t*_ is selected with the softmax exploration strategy, namely there is a unique temperature parameter $T_{s_{t}}$ for each state **s**_*t*_, which is decreased according to the number of visits to **s**_*t*_. Finally, *α* is a predefined learning rate.

Algorithm 1 One loop iteration of the user model-based learning procedure of B . Here, *t* is the current time step, *CS*_*t*_ the current user score, and U a uniform distribution.

1: **function** Q-iteration( $t,k,l,Q,\gamma ,\alpha ,s_{t},T_{s_{t}},CS_{t}$ )
2: $a_{t}∼\frac{e^{Q\left(s_{t},a_{t}\right)/T_{s_{t}}}}{\sum_{i=1}^{l+2}e^{Q\left(s_{t},a_{i}\right)/T_{s_{t}}}}$

3: *L*_*t*+1_, *F*_*t*+1_ ← *a*_*t*_(**s**_*t*_)
4: *PS*_*t*+1_ ← *CS*_*t*_
5: **s**_*t*+1_ ← (*L*_*t*+1_, *F*_*t*+1_, *PS*_*t*+1_)
6: $p\left(\text{success}|s_{t+1}\right)\leftarrow F_{k}^{p}\left(s_{t+1}^{p}\right)$

7: $O_{t+1}\leftarrow \left\{\begin{array}{cc} 1\; & p\left(\text{success}|s_{t+1}\right)\geq U\left(0,1\right)\\ -1\; & \text{otherwise} \end{array}\right\}$

8: $RE_{b,t+1}\leftarrow \left\{\begin{array}{cc} L_{t+1}\; & \text{if }O_{t+1}=1\\ -1\; & \text{if }O_{t+1}=-1 \end{array}\right\}$

9: $E_{t+1}\leftarrow F_{k}^{e}\left(s_{t+1}^{e}\right)$

10: *R*_*a*_(**s**_*t*_) ← *F*_*r*_(*RE*_*b*,*t*+1_, *E*_*t*+1_)
11: $Q\left(s_{t},a_{t}\right)\leftarrow Q\left(s_{t},a_{t}\right)+\alpha \left(R_{a}\left(s_{t}\right)+\gamma \underset{a}{\max}Q\left(s_{t+1},a\right)-Q\left(s_{t},a_{t}\right)\right)$

12: *CS*_*t*+1_ ← *L*_*t*+1_ ⋅ *O*_*t*+1_
13: **return** *Q*

### 2.6 Improvements of the robot behaviour model

Due to the properties of Q-learning, which is an off-policy learning algorithm, the training of the robot behaviour model might be relatively slow and take a lot of iterations. This is caused by the necessity for performing an exploration procedure in order to visit as many state-action pairs as possible. To improve the convergence speed of the algorithm, we investigate two possible improvements, namely i) policy pretraining and ii) learning from guidance.

#### 2.6.1 Policy pretraining

Policy pretraining means that the policy is not trained from scratch, but is initialised with a policy learned with another user model. This has the purpose of reducing the effort required for collecting user model data, as it can enable behaviour model training with *h* < *k* user models; additionally, pretraining can be used to improve the policy convergence speed, as shown in Tsiakas et al. (2016). For this reason, we investigate this technique as a possible improvement of the Q-learning-based algorithm above. Concretely, this means that, instead of starting the training procedure with a Q-value table initialised with zeros and then training on a user model $M_{k_{1}}$ (which would be the case if we start training from scratch), the table is initialised with the values obtained from the training with a user model $M_{k_{2}}$ .

#### 2.6.2 Learning from guidance

Learning from guidance can also improve the convergence speed of a policy (Tsiakas et al., 2016) and additionally allows expert knowledge to be incorporated in the system. We particularly investigate a *mistake correcting* technique (Torrey and Taylor, 2013), where a supervisor is advising the robot system exclusively on mistakes; this means that the robot needs to announce its intended action in advance so that the supervisor can correct it if necessary. To integrate mistake correcting in our learning algorithm, we use the *control sharing* method in Knox and Stone (2012, 2010), which fuses the supervisor’s knowledge with the reward that the robot can perceive directly from the environment; this can be interpreted as a way of guiding the robot’s action exploration. In the control sharing method, the probability of selecting an action that is suggested by the supervisor is $P\left(a_{t}=\underset{a}{\mathrm{arg max}}\hat{H}\left(s_{t},a\right)\right)=\min\left(\delta ,1\right)$ (otherwise, the agent’s own policy is used for action selection). Here, *δ* is a so-called *combination parameter*, which is annealed by a predefined factor, and $\hat{H}\left(s_{t},a_{t}\right)$ is the policy of the supervisor. We use control sharing rather than alternative methods, such as Q-augmentation (Tsiakas et al., 2018a) or reward shaping, as it can be used to enforce the inclusion of the supervisor’s actions in the robot’s behaviour policy; with the other two methods, the supervisor’s actions become more likely, but they are not guaranteed to be enforced.

Control sharing is straightforward to adapt to our framework so that our critical requirement—the robot can perform only an action that is approved by the supervisor—can be met; this can be done by making the combination parameter constant, such that *δ* ≥ 1. Algorithm 2 is a modified version of Algorithm 1 with the addition of the adapted control sharing combination technique. Here, it should be mentioned that ignoring external signals as a reward can be disadvantageous. In Senft et al. (2017), if the supervisor allows a wrong action to be executed by mistake, this action will be rewarded positively and would need to be corrected later. The problem becomes even more serious if the number of mistakes made by the supervisor is significant, as it means that the number of required corrections would also increase. In our model learning evaluation, we investigate how the probability of supervisor mistakes affects the learning progress of the behaviour model.

Algorithm 2 One loop iteration of the learning procedure of B with guidance. Lines 2–5 show the part that is responsible for control sharing.

1: **function** ModelGuidance( $t,k,Q,\hat{H},\gamma ,\alpha ,s_{t},CS_{t}$ )
2: $a_{t}=\underset{a}{\mathrm{arg max}}Q\left(s_{t},a\right)$

3: $a_{c}=\underset{a}{\mathrm{arg max}}\hat{H}\left(s_{t},a\right)$

4: **if** *a*_*t*_ ≠ *a*_*c*_ **then**
5:    *a*_*t*_ ← *a*_*c*_
6: Lines 3–13 of Algorithm 1

## 3 Results

### 3.1 Model learning evaluation

For the purpose of training user models, we require data collected from real users. In this section, we first explain the data collection process and then present results for training user models and their associated behaviour models. We explain the preprocessing procedure of the collected data, which is necessary for training user models. We then i) present the results for training behaviour models with the learned user models, ii) show how reward shaping can improve the quality of a policy, and iii) demonstrate how the policy convergence speed can be improved by applying policy transfer and learning from guidance. We also present the results from a conducted survey, whose main aim was to collect the experimental participants’ subjective point of view about the robot-supported sequence learning game. Extended results are presented in the Supplementary Material.

#### 3.1.1 Experimental setup

**FIGURE 2:**
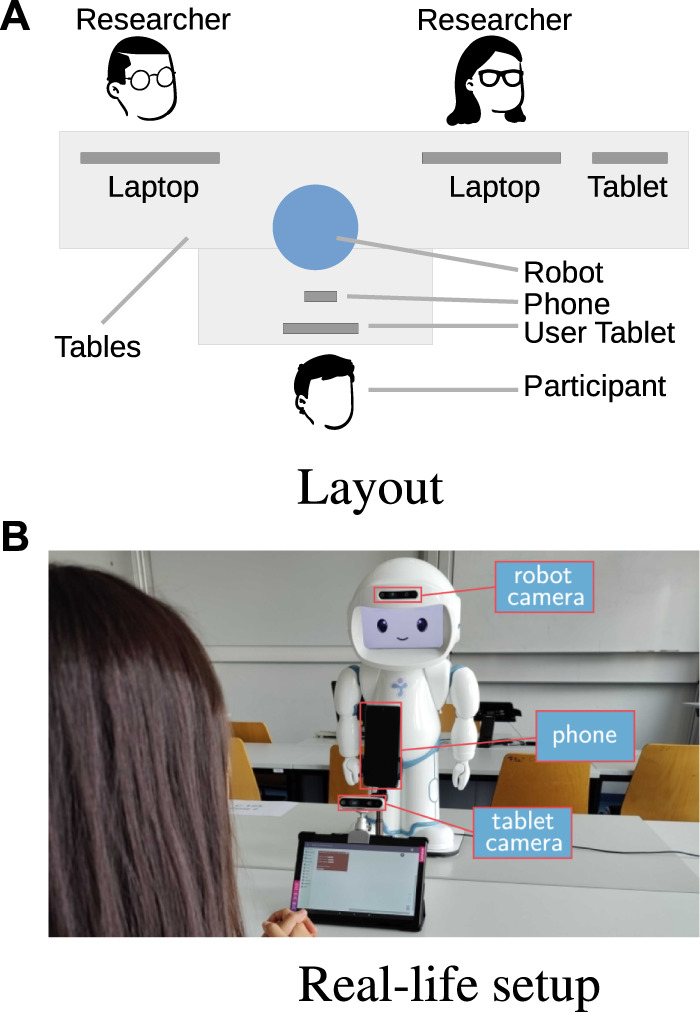
Experimental setup. The participant sits in front of the robot and plays the sequence learning game by interacting with the tablet. The robot estimates the participant’s engagement during the activity using its built-in camera. **(A)** Layout. **(B)** Real-life setup.

To evaluate the feasibility of the proposed user models and robot behaviour model, we collected data from 20 adult participants who played the game described in Section 2.1. The setup is depicted in Figure 2.

The participants were university students and research staff members; all of them had prior experience with robots, but some of them had never interacted with QTrobot. During the experiment, data were recorded from the robot’s sensors, a camera placed on top of the user tablet, and a smartphone placed between QTrobot and the tablet; for this work, we only used the data recorded from the robot’s RealSense camera^13^ ^14^. The therapist tablet was used by one of the researchers, while participants only interacted with the user tablet. Each participant completed one session of the game. Before starting, each participant was provided with a verbal explanation of the game. Within a session, participants had to memorise randomly generated sequences of each difficulty level, such that there were game stages when no feedback was provided and stages when feedback was provided after solving consecutive sequences of the same length. The purpose was to investigate the participants’ performance in the game and engagement during the interaction with the robot, but also their reactions to the feedback given by the robot. For this purpose, for each participant, we collected the game performance, estimated engagement scores, and timings of recreating the sequences on the tablet. The collected data were used for learning user and behaviour models as in Section 2.

#### 3.1.2 Survey

At the end of the data collection experiment, every participant was asked to fill out a survey, whose main aim was to show the participants’ subjective point of view about their engagement, game performance during the game, and game difficulty. Users could answer questions with the use of a three-point Likert scale. The same questions were asked for all of the game difficulty levels. We also asked for a written explanation about the reasons for each participant’s engagement, disengagement or being in a neutral state, for every difficulty level. All the survey questions were inspired by Tsiakas et al. (2018a).

**FIGURE 3:**
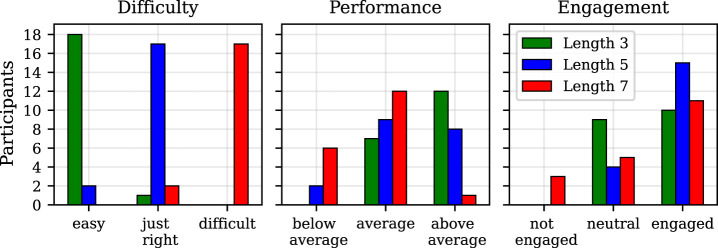
Results of the survey conducted after the data collection experiment. The diagrams show the participants’ own perception of the difficulty of the activities, their performance in the three difficulty levels, and their engagement for each difficulty level.

The results of the survey (answers with the three-point Likert scale) are depicted in Figure 3 ^15^.

Based on Figure 3, it can be stated that around 95% of participants considered the sequences of length 3 as easy, around 90% voted that a length of 5 is just right, and the same number of participants claimed that length 7 is too difficult. These results suggest that all of the participants had similar skills in memorising sequences; additionally, we confirmed that the hypothesis about the perception of the chosen lengths, based on which the game was designed, seems to hold. Regarding the participants’ opinions about their own performance during the game, all of them reported having average or above average performance for the shortest sequences; however, for the length of 5, more participants reported performing as average and some even below average. From this, it is visible that the participants were reporting worse performance with increasing difficulty. When it comes to the engagement of the participants, around 47% reported to be neutral and 53% to be engaged for the easiest sequences. It is visible that more users were engaged (around 79%) for the more difficult sequences (length 5); however, for the most difficult sequences, the number of participants that reported to be engaged is lower, but higher for those that claimed to be neutral or not engaged.

#### 3.1.3 Estimated engagement

**FIGURE 4:**
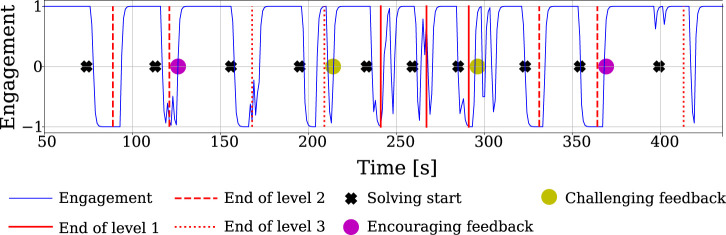
Expected engagement in one session for one of the participants. The black crosses are times when the user had to recreate a sequence on the tablet; the red lines mark the ends of game stages (with a certain *L*), which are followed by either the start of a new sequence or robot feedback given to the user.

An example evolution of the estimated engagement score during an interaction with one representative participant is shown in Figure 4. In particular, we show data from a user whose engagement value is as expected, namely the engagement is generally high when the robot is talking to the user (after the participant finishes solving a sequence) and decreases when the participant is asked to recreate the sequence on the tablet (as they had to look down at the tablet instead of at the robot). It is important to mention that some users did not behave as expected due to environmental disturbances or the way they were focusing on the robot; for instance, some users preferred to listen to the robot with closed eyes rather than look at it, which affects the engagement estimate^16^.

#### 3.1.4 User model training

**FIGURE 5:**
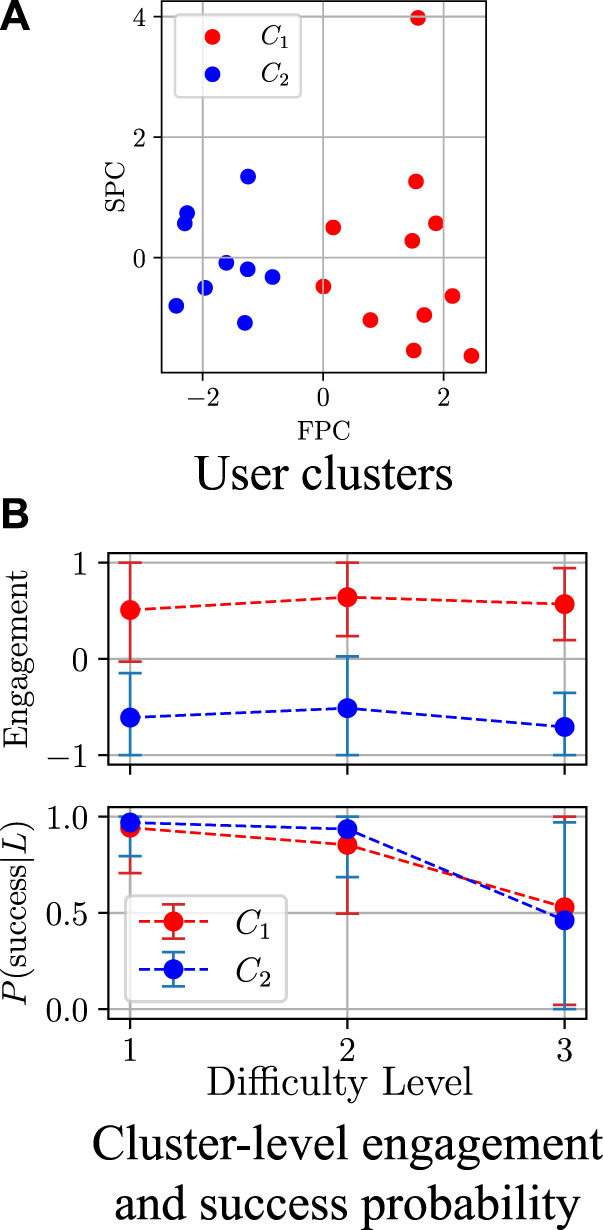
Clustering results (FPC and SPC stand for first and second principal components, respectively). One of the clusters represents users with high engagement, while the other represents users with lower engagement. The users in both clusters have comparable performance in the three difficulty levels. **(A)** User clusters. **(B)** Cluster-level engagement and success probability.

From the collected data, we created 20 vectors **u**_*j*_ as in Eq. (3), projected them onto a 2D space using PCA^17^, and clustered them into *c* = 2 groups (Figure 5A) using K-means clustering. It should be noted that the assignment of a user to a specific cluster represents their skill level on the day of the experiments; this level may vary and improve over time. We selected the number of clusters *c* based on our observations of the participants’ behaviour during the data collection; in our case, |*C*_1_| = 11 and |*C*_2_| = 9 participants, such that each cluster represents users that share a similar behaviour^18^. For each difficulty level *L* and user cluster *C*_*k*_, Figure 5B shows the mean and standard deviation values for the engagement *E* and the probability of success given a certain difficulty level *P*(success|*L*). Based on the results, it can be seen that *C*_1_ and *C*_2_ are similar with respect to *P*(success|*L*), but they significantly differ when it comes to *E*, as users belonging to *C*_1_ show a much higher level of engagement in the interaction with the robot than those in *C*_2_. After grouping the users, we calculated *P*(success|**s**) and the mean expected engagement (given success or failure in solving the sequence) for each cluster and interaction state **s**. Then, we trained four GP models, namely *F* ^*p*^ and *F* ^*e*^ for each cluster, and thus created two user models, $M_{1}$ (for *C*_1_) and $M_{2}$ (for *C*_2_).

**FIGURE 6:**
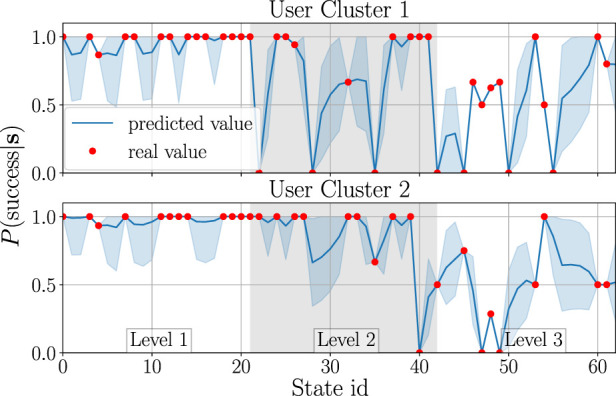
GP models used for creating *F* ^*p*^. The real value stands for the probability of solving the sequence correctly for each user cluster; the predicted value is the probability obtained from the GP. The envelopes represent one standard deviation from the mean estimate.

**FIGURE 7:**
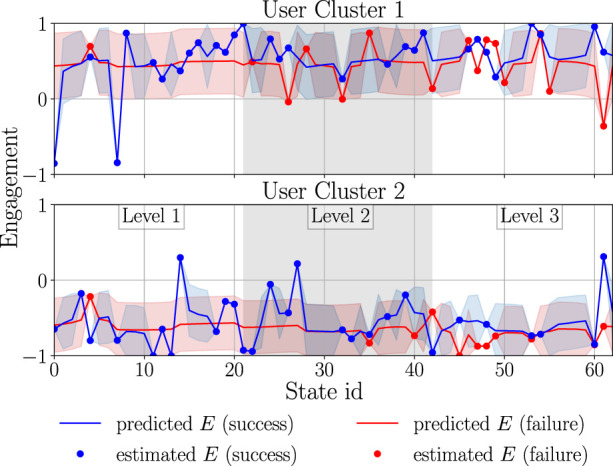
GP models used for creating *F* ^*e*^. The estimated engagement *E* stands for the average expected engagement calculated from all users in a given cluster, given that they solved the sequence correctly or incorrectly. The predicted engagement *E* is the output of the GP given a user’s failure or success in solving the sequence. The envelopes represent one standard deviation from the mean estimate.

Figure 6 and Figure 7 visualise the GP estimates of *P*(success|**s**) and the mean expected engagement *E*, respectively, for both clusters. Here, the GP was applied with the rational quadratic kernel (Rasmussen and Williams, 2006), which was selected experimentally.Due to space limitations, the *x*-axis shows state ID numbers instead of full state tuples^19^; however, for easier interpretation, the states are grouped into the corresponding game difficulty levels. As can be seen, the behaviour of the GP for unseen states is stable; this is desired, as participants were usually showing stable behaviour (mostly engaged or disengaged) during an interaction with the robot. Considering Figure 6, it may seem as if engaged participants should have a higher success rate in the game than disengaged participants. It is, however, important to mention that users may be focusing on the robot’s prompts while not looking at the robot at all—for instance, they may close their eyes while memorising sequences; this is why some disengaged users may outperform the engaged ones. Nevertheless, even though there may not be a direct relation between game performance and engagement, engagement is still an important factor which can enhance the behaviour model learning, as shown in section 3.1.5.

#### 3.1.5 Behaviour model training

After training the user models, we learned behaviour models as described in Section 2.5. Here, we investigate different approaches for calculating a reward for policy learning so that i) the engagement and game performance for *C*_1_ and *C*_2_ are maximised, and ii) longer sequences to maintain the interest of a user are enforced. We try to improve the policy convergence speed as well, following the concepts introduced in Section 2.6, namely i) using a pretrained policy and ii) applying learning from guidance. Given $M_{1}$ and $M_{2}$ , we trained two behaviour models as described in section 2.5. Similar to Tsiakas et al. (2018a), we set a small learning rate *α* = 0.05 to minimise instability under noisy observations. We also set a big discount factor *γ* = 0.95 so that the algorithm gives high importance to future rewards. We set the initial value of the temperature parameter $T_{s_{t}}$ (for each state) to 300 so that all actions are considered equal at the start of training.

##### 3.1.5.1 Engagement and performance score influence on policy learning

**FIGURE 8:**
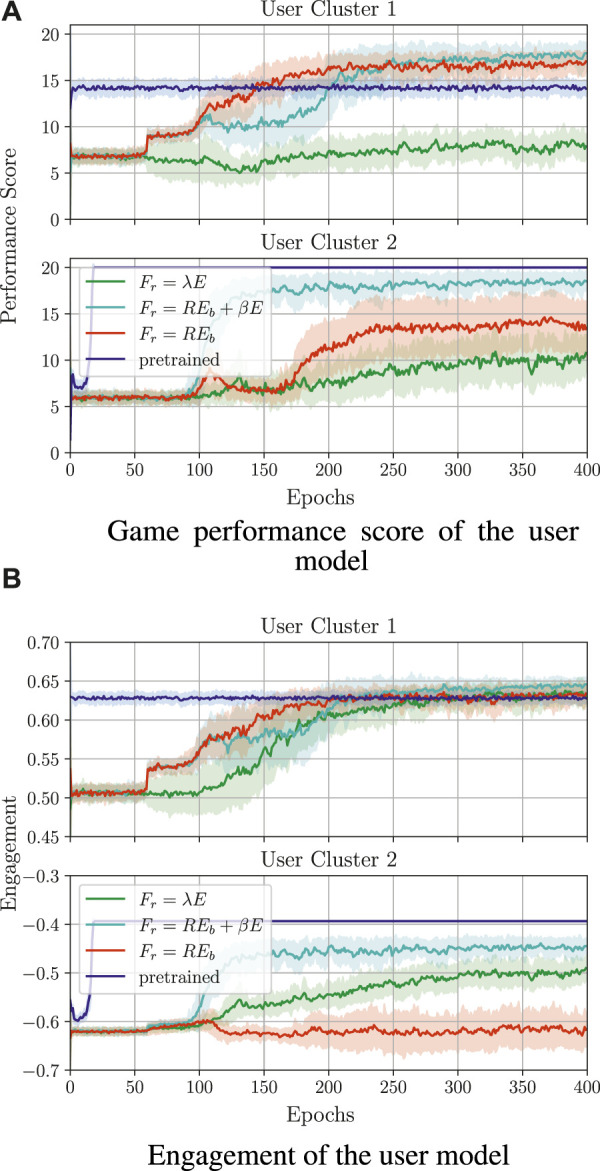
Comparison of different reward functions and policy transfer for training behaviour models. A reward function that combines both engagement and activity information generally leads to the highest training reward when a behaviour policy is trained from scratch. Policy transfer significantly improves the convergence speed, but the quality of the initialising policy has a clear effect on the performance of the trained policy (here, policy transfer was performed with *F*_*r*_ = *RE*_*b*_ + *βE*, with *β* = 3). **(A)** Game performance score of the user model. **(B)** Engagement of the user model.

To find an appropriate reward *R*_*a*_(**s**_*t*_), we compared three different candidates for the function *F*_*r*_ (as defined in section 2.5), namely i) *F*_*r*_ = *RE*_*b*_, ii) *F*_*r*_ = *RE*_*b*_ + *βE*, and iii) *F*_*r*_ = *βE*. We particularly aimed to check what influence *RE*_*b*_ and *E* have on the quality and speed of the policy convergence for both user models. The hyperparameter *β* depends on the range of values of *RE*_*b*_ and *E* and was selected empirically; in our case, *β* = 3. The average results of the training procedure (over 30 runs) are shown in Figure 8. In the figures, one training epoch is equal to 100 sessions of a sequence learning game, where each session means that the user has to solve *ω* = 10 sequences. The performance score (Figure 8A) stands for the mean accumulated activity score (accumulated in one session and averaged over the epoch). As shown in Figure 8A, calculating the reward by combining both *E* and *RE*_*b*_ helps in quick personalisation of the game difficulty for $M_{2}$ , but is not more advantageous in comparison to using only *RE*_*b*_ for $M_{1}$ . On the other hand, when using *F*_*r*_ = *βE*, the trained policy gives the worst results with respect to the performance score. We obtained different results when evaluating the training process with respect to the user’s engagement. In Figure 8B, it can be noted that, for $M_{1}$ , all three versions of *F*_*r*_ lead to similar results, while, as expected, the lowest engagement for $M_{2}$ is obtained when the engagement information is ignored in the reward. The policy training seems to have better results when *F*_*r*_ = *βE* and can be meaningfully improved when both *RE*_*b*_ and *E* are considered. Based on the aforementioned results, it can be concluded that adjusting the task difficulty by combining the engagement and activity performance score for computing *R*_*a*_(**s**_*t*_) can help in increasing the engagement; this is in line with Tsiakas et al. (2016).

##### 3.1.5.2 Influence of policy pretraining on the convergence speed

To increase the policy convergence speed, we also attempted policy transfer (Figure 8), namely the Q-table for one cluster was initialised with the Q-table that was trained on the other user model. The initial policy was chosen (out of 30 learned policies) based on the highest average return value in the last pretraining epoch. Here, training was performed with *F*_*r*_ = *RE*_*b*_ + *βE* and an exploitation-only-based strategy, as exploration might lead to undesired robot actions during real-life therapeutic scenarios.

Analysing the performance score and the engagement, it can be seen that the initialisation of the lower engagement policy ( $M_{2}$ ’s) with the higher engagement policy ( $M_{1}$ ’s) improves both the speed and the quality of personalisation, such that the initial policy can not only adapt, but it also gives slightly improved results over the user-specific policy; this seems to indicate convergence to a local minimum for the policy trained from scratch, but it also indicates that a proper initialisation can provide useful inductive bias for the learning process, which is consistent with results in the transfer learning literature (Ramon et al., 2007 Tsiakas et al. (2016)). When initialising $M_{1}$ ’s policy with $M_{2}$ ’s policy, however, the engagement and performance score remain unchanged over the entire training procedure. This may be caused by obtaining mainly positive rewards during training, as $M_{1}$ usually outputs a positive *E* (Tsiakas et al., 2016).

##### 3.1.5.3 Enforcing longer sequences

So far, the presented results seem to be promising; however, it is also important to mention that, based on the training data, the created user models $M_{1}$ and $M_{2}$ output relatively high success probabilities for sequences of lengths 3 and 5, but the probabilities are lower for the longest sequences. Thus, to maximise the game performance, the behaviour model may learn to suggest only sequences of length 5, as longer sequences are less likely to be solved successfully.

With the *RE*_*b*_ score introduced in section 2.5, the robot indeed learns to choose sequences of 5. In a real-life interaction, the user may get bored of getting sequences of the same length; thus, to make the game less monotonous and more challenging, we enforce the selection of more difficult sequences during the game. To achieve this goal, we changed the calculation of the *RE*_*b*_ component of *F*_*r*_, such that we conducted experiments with two alternatives, namely

$$RE_{d,t}\leftarrow \left\{\begin{array}{cc} 2L_{t}\; & \text{if }O_{t}=1\\ -1\; & \text{if }O_{t}=-1 \end{array}\right.,RE_{s,t}\leftarrow \left\{\begin{array}{cc} L_{t}^{2}\; & \text{if }O_{t}=1\\ -1\; & \text{if }O_{t}=-1 \end{array}\right.$$ (6)

As a result of these changes, the agent will obtain a larger reward if the user correctly solves longer sequences. In order to obtain satisfying results, we also adjusted the parameter *β* accordingly. When using *RE*_*d*_ with *β* = 5 for learning $M_{1}$ , the number of states in which the most difficult sequence is preferred increased from 4 to 6 as compared to the case in which *RE*_*b*_ was used; additionally, the robot became slightly more interactive, as it gives not only challenging feedback, but also encouraging one. For the cluster with disengaged users *C*_2_, the number of states where the sequence of length 7 is chosen increased from 1 to 2; here, the robot is more interactive as well and provides feedback for more states. Applying *RE*_*s*_ with *β* = 8 significantly increased the difficulty of the game: for $M_{1}$ , a sequence of length 7 is chosen in 14 states, and in 7 states for $M_{2}$ .

**FIGURE 9:**
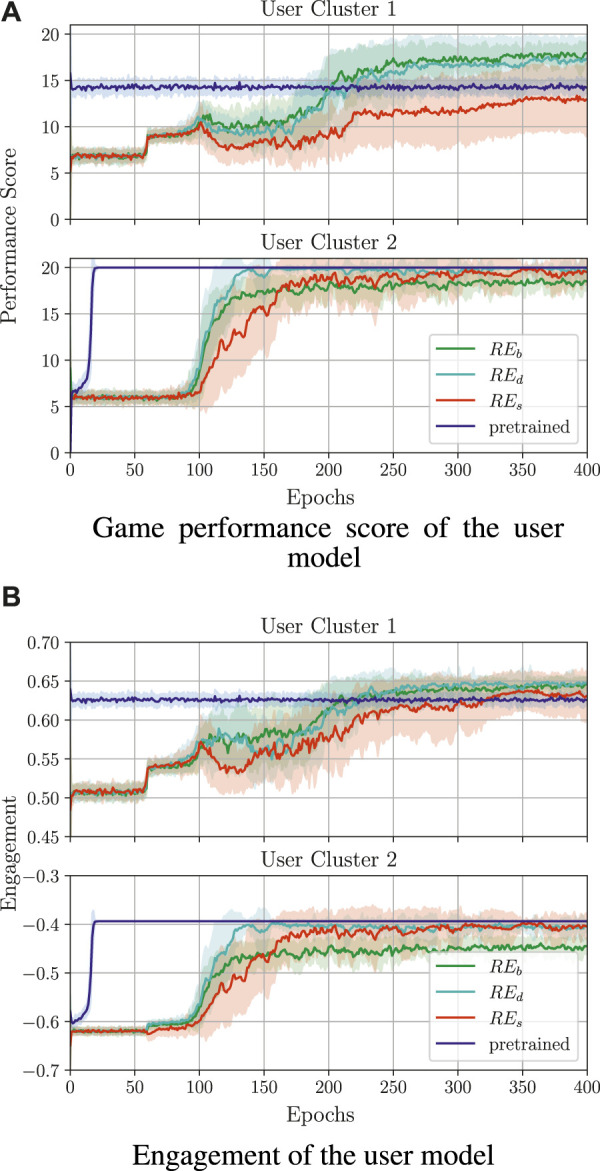
Policy training results for different *RE* variants and *β* values in order to enforce longer sequences. Here, *RE*_*b*_ is a reward as in section 3.1.5.1, *RE*_*d*_ doubles the reward for correct answers, and *RE*_*s*_ squares the reward for correct answers. In all cases, the activity reward was combined with the engagement as above, namely *F*_*r*_ = *RE*_*x*_ + *β*_*x*_ *E*, *x* ∈ {*b*, *d*, *s*}, *β*_*x*_ ∈ {3, 5, 8}; the pretrained policy was learned using *RE*_*b*_ and *β* = 3. **(A)** Game performance score of the user model. **(B)** Engagement of the user model.

The results of the policy training with different *RE* variants are shown in Figure 9; in particular, the results were obtained for *F*_*r*_ = *RE*_*x*_ + *βE*, *x* ∈ {*b*, *d*, *s*}, as this reward function was shown to perform best in the above evaluation. Here, as before, we present the results for policy transfer, but only for *RE*_*d*_, as this gave the best training results while keeping the length of the sequences more challenging. As can be seen, applying *RE*_*d*_ and *RE*_*s*_ positively affects the performance score and engagement of $M_{2}$ , which reach 20 and −0.4, respectively, which are slightly better than for *RE*_*b*_. On the other hand, there is not much difference in the game performance and engagement for $M_{1}$ between *RE*_*b*_ and *RE*_*d*_. When applying *RE*_*s*_, the results suggest that giving the user more difficult tasks during the game does not necessarily lead to a higher performance score nor engagement. Finally, learning with the pretrained policy for *RE*_*d*_ gives similar results as for *RE*_*b*_.

##### 3.1.5.4 Learning from guidance

For exploring learning from guidance, we created simulated supervisor policies $\hat{H}\left(s_{t},a\right)$ based on the policies learned above, namely the policy with the highest average return value in the last training epoch (out of 30 learned policies) is used as the supervisor policy. In real supervision, a supervisor may potentially make correction mistakes; to reflect this aspect in the evaluation, we investigate noisy supervisor policies, which are simulated by selecting random actions with different probabilities (*P*(*err*) = 0.1 and *P*(*err*) = 0.2). We conducted the experiments in this section with *F*_*r*_ = *RE*_*d*_ + *βE*, as the obtained policy with this reward increases the user’s game performance and engagement, and also enforces longer sequences during the game; we set the parameter *β* in *F*_*r*_ to 3^20^.

**FIGURE 10:**
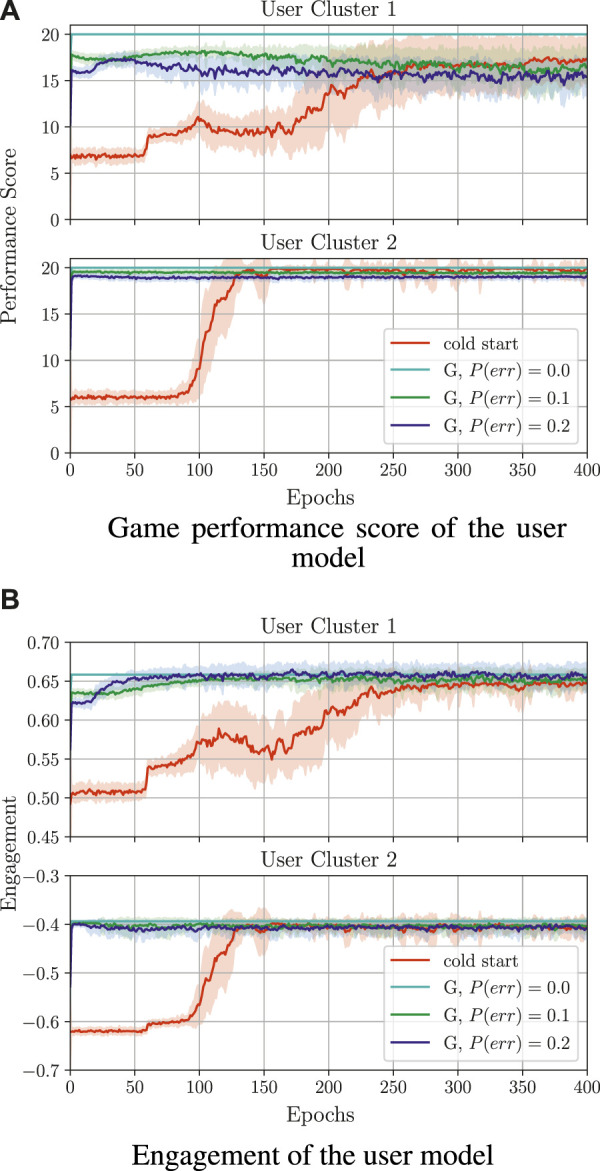
Comparison of training behaviour models from guidance (denoted by G) with different probabilities of the supervisor making a mistake. Here, cold start refers to a policy trained from scratch without supervisor guidance. All guidance-based policies were trained using the reward *F*_*r*_ = *RE*_*d*_ + *βE* and *β* = 3; the cold start policy was trained with *β* = 5. **(A)** Game performance score of the user model. **(B)** Engagement of the user model.

The training results for learning from guidance are depicted in Figure 10. Here, the shown performance score and engagement for each sequence in the game are obtained by simulating the execution with an action selected from the policy that is continuously learned^21^. As a baseline for the training quality, we compare the policies learned from guidance with a policy learned from feedback (denoted as *cold start*).

For both clusters, learning from guidance is successful and the performance score and engagement are close to the values obtained by the supervisor’s policy (Figure 10). In particular, with *P*(*err*) = 0.0, the optimal performance and engagement (as obtained in the case of learning from feedback) is already reached at the beginning of the guidance-based training, namely a performance score of around 20 for both $M_{1}$ and $M_{2}$ , as well as an engagement of around 0.65 and −0.4 for $M_{1}$ and $M_{2}$ , respectively. For bigger *P*(*err*), the performance score and engagement vary more during the training.

**FIGURE 11:**
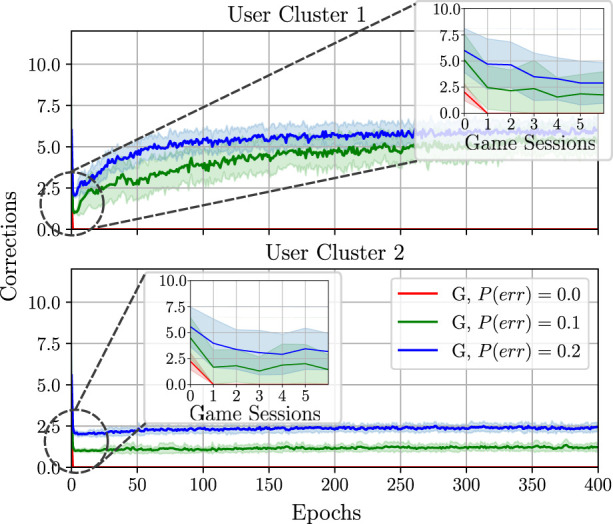
Comparison of training behaviour models from guidance (denoted here by G) with different probabilities of the supervisor making a mistake *P*(*err*). All policies were trained using the reward *F*_*r*_ = *RE*_*d*_ + *βE* and *β* =3.

Another good measure of training progress in learning from guidance is the number of corrections that the supervisor needs to do over time. Given that the supervisor aims to increase the user’s engagement and game performance, and they only select a small number of inappropriate actions, after a specific time, the agent should be able to select the correct actions on its own. Figure 11 shows the short-term corrections (accumulated over one session in the first seven game sessions) as well as the long-term corrections (mean accumulated corrections over one session and averaged over the epoch).

Here, it can be seen that, with increasing *P*(*err*), the algorithm may need to do more corrections over time to learn successfully, namely the larger the value of *P*(*err*) is, the longer the training process takes. This may negatively affect the long-term training behaviour, such that, after a long period of time, the number of necessary corrections from the supervisor may increase (for *C*_1_) or remain unchanged (for *C*_2_) instead of being reduced to zero.

### 3.2 User study

Our final experiment is concerned with a small-scale real-life feasibility evaluation of the developed behaviour model. For this purpose, we conducted a study with six participants, three of which already participated in the data collection study explained in Section 3.1. The experimental setup was similar to the one presented in Figure 2, but only the robot’s head camera was used here. This experiment compared three conditions in which the sequences were i) randomly generated (as in the data collection study) as a baseline, ii) chosen based on a policy pretrained in the simulation (as in Section 3.1.5.3), and iii) chosen based on a policy trained on the fly using learning from guidance^22^. The study was single-blind, namely the evaluated mode was not known to the participants during the experiment^23^. At the end of each condition, each participant was asked to fill out a survey with three questions^24^. The main aim of this survey was to collect the user’s opinions about the different experimental conditions and their perception of the robot in each evaluation mode.

In the evaluation of the policy learned with the user models, we consider two cases depending on whether the participant took part in the data collection study. For participants that took part in the study, we trained a policy on the user model learned on the corresponding user cluster^25^. For the other users, we used the policy trained on $M_{2}$ , which represents users that are mostly disengaged. This policy selects easier sequence lengths in comparison to the policy trained on *M*_1_, which prevents users from becoming disengaged because of too difficult tasks.

For the randomised condition and the condition in which a pretrained policy was used, only one session was performed. In the learning from guidance condition, each user played four game sessions, where the first three sessions were used for training (the supervisor was allowed to guide the robot)^26^ and the last one was a testing session (no guidance was allowed, namely the robot followed the policy learned up to that point). For learning from guidance, we used the same reward function as for training with the user models, namely *F*_*r*_ = *RE*_*d*_ + *βE*; however, as explained in Section 3.1.5.4, we also tuned the parameter *β* to properly adjust the reward function.

Before presenting the results of this study, it should be noted that, due to the small sample size, the results can only show a tendency of the model to adapt to individual users, but a larger user study is required to demonstrate the significance of personalisation on the engagement and learning success.

#### 3.2.1 Behaviour model evaluation

**FIGURE 12:**
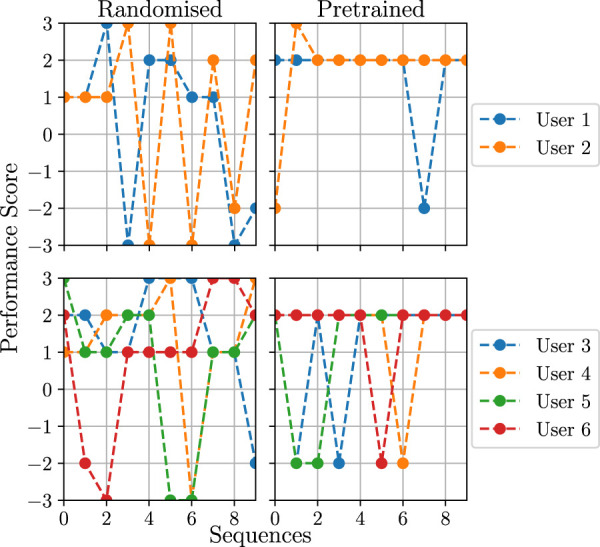
Results of the user study for the randomised and policy transfer conditions. The users are split into multiple diagrams in order to improve the readability. Users one to three are the ones that also took part in the data collection study.

**FIGURE 13:**
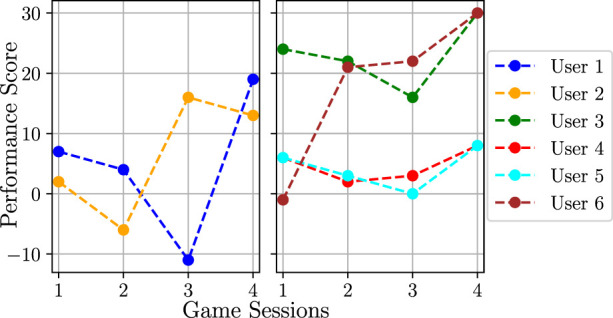
Cumulative performance score (over each session) for the user study in the learning from guidance condition. The used reward function is *F*_*r*_ = *RE*_*d*_ + *βE*, where *β* = 3 for users 1 and 2 (left plot) and *β* = 5 for the remaining users (right plot). Users one to three are the ones that also took part in the data collection study.

The results of the evaluation with respect to the users’ game performance (activity score obtained for each sequence as defined in Section 2.4) are presented in Figure 12 and Figure 13.

As shown in Figure 12, for the randomised condition, the task difficulty chosen by the robot does not follow any logical pattern, which results in a varying game performance. This is especially visible for user 1; in particular, between sequences 4 and 7, it can be seen that the difficulty is decreasing, even though the user is solving the sequences correctly. It should be noted that, in some cases, the same difficulty level is repeated twice, as the robot chooses an action of giving feedback to the user, which implies repeating the same sequence length. In the condition where the policy trained on the user model was used, the robot mostly chooses the same difficulty level. The consequence of this is either explicitly providing a sequence of length 5 or selecting a feedback action, which by definition provides verbal feedback and repeats the same sequence length. This means that the behaviour model found these actions as the ones that maximise the game performance and engagement score based on the user models^27^.

**FIGURE 14:**
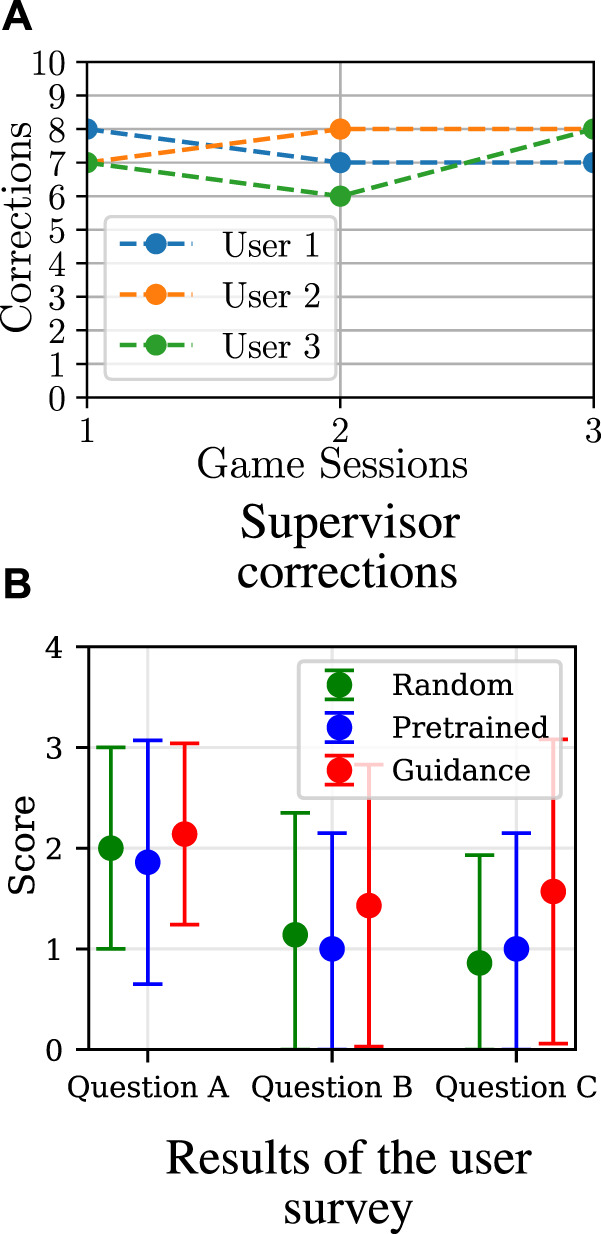
Results of the real-life evaluation. In the user study, there seems to be a slight preference for the robot when trained using guidance; however, due to the small number of participants, this should not be seen as a statistically significant result. **(A)** Supervisor correction. **(B)** Result of the user survey.

The results of the learning from guidance condition are depicted in Figure 13, where the accumulated performance score for all four sessions is visualised. Here, it can be seen that, for all users, the score varies in the first three sessions, which is a result of the supervisor intervening in the action selection process. In the fourth session, in which supervisor corrections were not allowed anymore, there is a visible performance improvement in comparison to the first session (at the beginning of the training procedure) for all users. This means that the model improved in choosing actions in order to increase the users’ game performance. For some users, however, the improvement of the accumulated score between the first and the last session is very small; this is particularly visible for users 5 and 6, such that it might be a result of *β* = 5 as discussed in Section 3.1.5.4. During the experiment, as shown in Figure 14A, no significant decrease in the required supervisor corrections over the training sessions was observed, which indicates that more game sessions may be required for the policy to converge.

#### 3.2.2 Survey

The answers to the survey questions were given with 5-point Likert scale, where 0 corresponds to an answer “not at all” and 4 for “extremely”. The survey results are depicted in Figure 14B. Here, it can be seen that the average score for all questions was the highest for the condition in which the robot was learning from guidance and continuously refining its policy; this is particularly visible for Question C. It should be noted that the answers of the participants were varied and, as a result, the standard deviations in Figure 10A are large; however, given the small number of participants, no statements about the statistical significance of these results can be made.

## 4 Discussion

In this work, we presented a reinforcement learning-based personalisation approach that increases the autonomy of a robot in the context of robot-assisted therapy. The proposed personalisation pipeline uses elements of both learning from feedback and learning from guidance, enabling both techniques to be used for behaviour model learning, either independently or in combination. For this purpose, we described a robot behaviour model that can be used to learn personalised robot policies (in terms of provided feedback and activity difficulty level) for groups of similar users. We also created user models from data collected during a study with 20 participants; these models incorporate engagement that is estimated based on visual features and were used for training personalised behaviour models. The proposed models were evaluated on a sequence memorisation game. From the evaluation, we can conclude that computing rewards based on both user engagement and activity performance generally increases the policy convergence rate. We also found that calculating the game performance part of the reward function as a double sequence length seems to be the most promising for learning a practically useful behaviour model, as it preserves a high engagement and performance score of the user, while also increasing the rate of selecting longer sequences during the game. To improve the policy convergence speed, we performed policy transfer experiments, where the policy for one user cluster was used to train the policy for another user cluster. This type of policy transfer can significantly improve the policy convergence speed, but may also lead to undesired results if the initialising policy or reward function are inappropriate. Finally, we conducted trial runs for learning from guidance, which leads to the fastest convergence speed and can indeed reduce the workload of the therapist by decreasing the number of necessary corrections. To check the potential practicality of the proposed behaviour model, we performed a small-scale real-user feasibility study under three conditions (randomised behaviour policy, policy learned based on user models, and policy learned from guidance). The results seem to suggest that the model is able to learn what actions to choose in order to increase a user’s engagement and game performance score, particularly when learning from guidance is used for policy learning; however, a large-scale study is needed to conclusively verify that observation.

There are various limitations of this work that we would like to discuss. Firstly, the full potential and importance of giving feedback to the user could not be explored in this study, as the game sessions were too short to capture how a user can get bored over a longer period of time and how appropriately the given feedback can change the engagement. Even if the game was long enough, it would be difficult to observe the long-term changes in the users’ engagement, as the trained engagement estimator often outputs a negative engagement score if a person is sitting too far from the robot, even if they are looking directly at the robot; this might be the reason why some users were constantly disengaged during the game, according to the used engagement estimation model. A better selection of the used features for estimating engagement as well as a manual annotation of the dataset used for training the model could potentially make the engagement model more accurate. With the aforementioned changes, it would still be difficult to evaluate the pure effect of the robot’s actions on each participant’s behaviour, as the participant was not alone in the room and could occasionally be distracted by external factors, such as unintentional distractions by the researchers. Due to the aforementioned flaws during the data collection and data preprocessing, the created user models may not accurately reflect the characteristics of the participants. Another drawback of the created user models is that they are static, which means that they do not encode the learning capabilities of each user, namely their increasing ability to memorise longer sequences over time. Ideally, the user models should be improved based on the ongoing interactions with the behaviour model; this may, however, lead to a complex dependency relation between the user model and the behaviour model, both of which would need to be continually updated.

In future work, we want to incorporate the behaviour model in activities used in the therapy of children with ASD, which would enable us to perform long-term evaluation with therapists and affected individuals. Additionally, one way to improve it would be the inclusion of a discretised engagement score into the state representation, similar to Senft et al. (2015a); this would increase the size of the state space and may make the learning problem more difficult, but would lead to a more complete representation of human-robot interaction scenarios. Finally, even though the focus of this work was on robot-assisted therapy, our intention for future work is to apply the proposed method in educational robotics contexts, such as where a teacher is providing guidance to a robot that is teaching a student a new language. Similarly, we would like to extend the method to human-robot collaboration scenarios, where a robot may provide assistance in activities that can be classified into different difficulty levels, for instance when users have varying levels of expertise.

## Data Availability

The raw data supporting the conclusion of this article will be made available by the authors, without undue reservation.

## Footnotes

1 Robots are attractive for this problem because children have been shown to find it more comfortable to interact with a robot than with a person (Robins et al., 2006).

2 The implementation can be found at https://github.com/migrave/migrave_personalised_behaviour_model.

3 We have presented a more detailed discussion of different personalisation techniques in (Stolarz et al., 2022b), including ones that are not directly relevant for this study.

4
https://xgboost.readthedocs.io

5 The training implementation can be found at https://github.com/migrave/migrave_models.

6 We make this assumption based on observations of users while interacting with the robot.

7 We particularly want to represent the characteristics of a given user group as accurately as possible, while also modelling the decision uncertainty; for this, we have found that a GP produces better results than a neural network or Support Vector Regression as used in Tsiakas et al. (2018a).

8 This mean is calculated only for periods when the user is supposed to be focused on the robot, for instance when the robot is talking to the user.

9 The number of principal components should ideally be chosen so that a predefined data variance is covered in the projection.

10 A positive or negative score is given for correctly or incorrectly solved sequences of a given difficulty level, respectively, and a 0 score is only used in the initial state, before a sequence length has been selected.

11 It should be noted that actions *a*_*l*+1_, *a*_*l*+2_ cannot be performed for the first sequence in a session as there is no context to give feedback.

12
*RE*_*b*_ is assigned −1 for an incorrect answer regardless of the difficulty level *L* in order not to discourage the robot from choosing a high *L*.

13 We recorded data from the camera on the tablet in order to estimate the engagement as seen from the tablet’s perspective while the user was reproducing the sequence on the tablet; this estimation is, however, not used in the current version of the system due to its low performance.

14 The smartphone was used to run the AffdexMe application: https://play.google.com/store/apps/details?id=com.affectiva.affdexme. AffdexMe can estimate engagement and was supposed to be used for cross-checking the estimates of our model, but the application was occasionally unable to detect the person, so its estimates are not used in this study.

15 It should be noted that only 19 participants completed the survey; one participant was the researcher responsible for the data collection, so we suspect that his subjective point of view would have been biased by the answers of the other participants.

16 A video illustrating the expected engagement estimates during the sequence learning game is available at https://youtu.be/-_CryYRz8DY.

17 We chose a 2D projection in the evaluation following Tsiakas et al. (2018a).

18 For a larger group of users, an automatic cluster selection may need to be performed, which would result in an optimal number of clusters (Schubert, 2023).

19 For instance, **s** ^*p*^ = (1, 0, − 1) denotes a sequence of difficulty level 1, no feedback given, and an incorrectly solved previous sequence of difficulty level 1. Each such state is assigned a unique ID in the shown diagrams.

20 In case of negative engagement, larger values of *β* can cause the reward to be zero or negative and thus slow down or even prevent any learning from happening. This is because the Q-values corresponding to the supervisor’s actions would obtain a negative reward, which can lead to the opposite results than expected, namely the agent will avoid actions selected by the supervisor.

21 Since we perform learning from guidance, the selected action is then corrected with the supervisor’s policy, such that the new engagement and performance score after the execution are used for the policy update (the Q-table entry corresponding to the supervisor’s action is updated).

22 In this mode, the researcher acts as a supervisor guiding the robot.

23 The involvement of users who participated in the data collection study may be considered as a source of bias in the evaluation, as they already have familiarity with the sequence learning game; however, considering the single-blind aspect of the study and given that our main objective was to investigate the participants’ performance and preferences with respect to the personalisation mode, we consider any such bias to be negligible. In addition, the two studies were conducted with a gap of several months; considering the engagement estimation, this means that the data distribution is not the same over the two studies.

24
*Question A*: How interactive was the robot? *Question B*: How much has the robot helped you perform to the best of your ability? *Question C*: If you played the game long enough, would the robot help you memorise longer sequences?

25 According to the numbering of the users in Figure 12, the corresponding user cluster was *C*_2_ for users 1 and 3 and *C*_1_ for user 2.

26 We trained the model for three sessions as, according to the experiments done with the user models, after three sessions, there should be a visible decrease in the number of required supervisor corrections.

27 From the performance point of view, this is reasonable because most users are indeed able to correctly solve sequences of length 5; however, continuously providing sequences of the same length is unlikely to lead to an enjoyable experience in general.
