# Supplementary material for: Learning-based personalisation of robot behaviour for robot-assisted therapy
Source: Front Robot AI. 2024 Apr 8;11:1352152. doi: 10.3389/frobt.2024.1352152 (PMC11033723; doi:10.3389/frobt.2024.1352152)
Supplement: Supplementary file 1 [file DataSheet1.PDF]

Supplementary Material

1 USER MODELLING

The reason why we decided to use a GP for creating $\mathcal{M}_1$ and $\mathcal{M}_2$ is that it could fit the collected data well and showed stable behaviour for unseen states. We came to the aforementioned conclusion after conducting an evaluation of five models: neural network (NN), Gaussian process (GP), random forest (RF), Support Vector Regressor (SVR) and AdaBoost (AB).[1] For each of these, we calculated the Root Mean Squared Error (RMSE) of the estimates:

$$RMSE = \sqrt{\frac{1}{N}\sum_{i=1}^{N}(y_i - \hat{y}_i)^2} \tag{S1}$$

where N stands for the total number of samples, $\hat{y}_i$ is the predicted value and y_i is the ground truth. We calculate the RMSE on the training data, as the purpose is to represent the specific data as accurately as possible. The RMSE for all five models is depicted in Table S1. It should be mentioned that the

Table S1. Root Mean Squared Error (RMSE) on the training dataset for various machine learning approaches for creating a user model

Model	Cluster	RMSE				
		GP	SVR	NN	RF	AB
F^p	C_1	2.89×10^{-10}	3.14×10^{-4}	3.91×10^{-2}	1.25×10^{-1}	1.37×10^{-1}
	C_2	3.49×10^{-10}	2.98×10^{-4}	9.27×10^{-3}	1.16×10^{-1}	8.40×10^{-2}
F^e	C_1	2.35×10^{-10}	2.83×10^{-4}	6.72×10^{-3}	1.59×10^{-1}	1.9×10^{-1}
	C_2	2.88×10^{-10}	2.94×10^{-4}	3.37×10^{-3}	1.36×10^{-1}	1.9×10^{-1}

input to the aforementioned machine learning models was not in the form of the state $\mathbf{s^p} = (L, F, PS)$ or $\mathbf{s^p} = (L, F, PS, O)$; instead, the variable F was replaced with a one-hot encoding $\mathbf{f}$ that represents categorical information as follows: $\mathbf{f} = (1,0,0)$ for $F = 0$, $\mathbf{f} = (0,1,0)$ for $F = 1$ and $\mathbf{f} = (0,0,1)$ for $F = 2$. Moreover, the values of L were scaled into the range between 0 and 1 and the values of PS were scaled into the range between -1 and 1. Before the training procedure, a z-score normalisation was performed on the vectors $(L, \mathbf{f}, PS)$. The GP was applied with the rational quadratic kernel (chosen in an experimental way) that can be expressed as

$$k(x_i, x_j) = \left(1 + \frac{d(x_i, x_j)^2}{2\alpha l^2}\right)^{\alpha} \tag{S2}$$

where α stands for the scale mixture parameter, l for the length scale and $d(\cdot,\cdot)$ is the Euclidean distance. The hyperparameters α and l were auto-tuned[2] for every F^e and F^p (the result parameters are depicted in Table S2). In the case of NN, F^p is a three-layer perceptron. Following the architecture in the publicly available implementation[3], the input layer consists of 20 neurons with a linear activation function; next, there is a 10-neuron layer (using hyperbolic tangent as an activation function) and a one-neuron output

[1] For implementing an NN, we used the TensorFlow library: `https://www.tensorflow.org`; the scikit-learn library `https://scikit-learn.org` was used for implementing the other models.

[2] Hyperparameter auto-tuning is provided by the scikit-learn library.

[3] The implementation can be found at `https://github.com/TsiakasK/sequence-learning-dataset`

Table S2. Parameters for the trained GP with the rational quadratic kernel

**Model**	**Parameter**	**Cluster**	
		C_1	C_2
F^p	l	2.79×10^{-1}	6.55×10^{-1}
	α	2.13×10^{-2}	2.04×10^{-2}
F^e	l	1.0×10^{-5}	5.02×10^{-3}
	α	6.83×10^{-3}	7.74×10^{-3}

layer with the sigmoid function. Additionally, it should be mentioned that all the layers are fully-connected. F^e is designed similarly; however, the hyperbolic tangent is used as an activation function for all the layers. Both NN models were trained for 10,000 epochs with a batch size of 8 and Adam optimizer. These options enable accurate representation of the training data, which is necessary for our use case.

Other models, namely SVR, RF and AB, were trained with the use of the grid search hyperparameter tuning method, such that the lowest RMSE on the training data is obtained. The hyperparameters found for SVR were as follows: RBF kernel function, $\epsilon = 0.001$ and $C_r = 10000$ for C_1 and $C_r = 1000$ for C_2. The RBF kernel function can be expressed as

$$k(x_i, x_j) = \exp\left(-\frac{d(x_i, x_j)^2}{2l^2}\right) \tag{S3}$$

where the symbols have the same meaning as in Eq. (S2). The length scale parameter l was chosen as a default parameter provided by the scikit-learn library.[4] The regularisation parameter C_r is inversely proportional to the strength of regularisation, thus it is has a high value. On the other hand, the parameter ϵ is very low to prevent large errors, as ϵ defines a margin of tolerance within which no penalty is given to errors. It is important to mention that a high C_r and low ϵ enable the model to overfit to the training data. When it comes to the RF model, the tuned hyperparameter was the number of used estimators N_e, which is as follows: F^p, C_1: $N_e = 100$; F^p, C_2: $N_e = 900$; F^e, C_1: $N_e = 700$; F^e, C_2: $N_e = 300$. We performed similar tuning of the AB model, for each N_e is as follows: F^p, C_1: $N_e = 800$; F^p, C_2: $N_e = 800$; F^e, C_1: $N_e = 500$; F^e, C_2: $N_e = 500$.

The trained regression models are shown in Fig. S1. Due to space limitations, the x-axis is annotated only with the state ID numbers; however, the order of states is similar as in the policy visualisation figures in section 2.2, namely the state with ID $= 0$ is $(3, 0, -3)$, while the state with ID $= 62$ is $(7, 2, 3)$. Only SVR is depicted here for comparison, as, according to Table S1, we obtained the smallest RMSE error for SVR and GP (presented in the article). For the training of the Q-learning RL model, we decided to use a GP, as its behaviour for unseen states is more stable. This is especially visible for Fig. S1 **(B)**, where SVR for C_1 for the predicted E (given the user made a mistake) significantly varies its output for the unseen states; such results may not be desired, as participants were usually showing stable behaviour (mostly engaged or disengaged) during an interaction with the robot. The undesired behaviour of SVR is also visible in Fig. S1 **(A)** for C_2, where $P(\text{success}|L)$ is drastically decreasing below 0.5 between the third and fourth datapoint, even though the inputs are the game states of the easiest difficulty level (sequence of length three), which were successfully completed by most of the participants.

[4] `https://scikit-learn.org/stable/modules/generated/sklearn.svm.SVR.html`

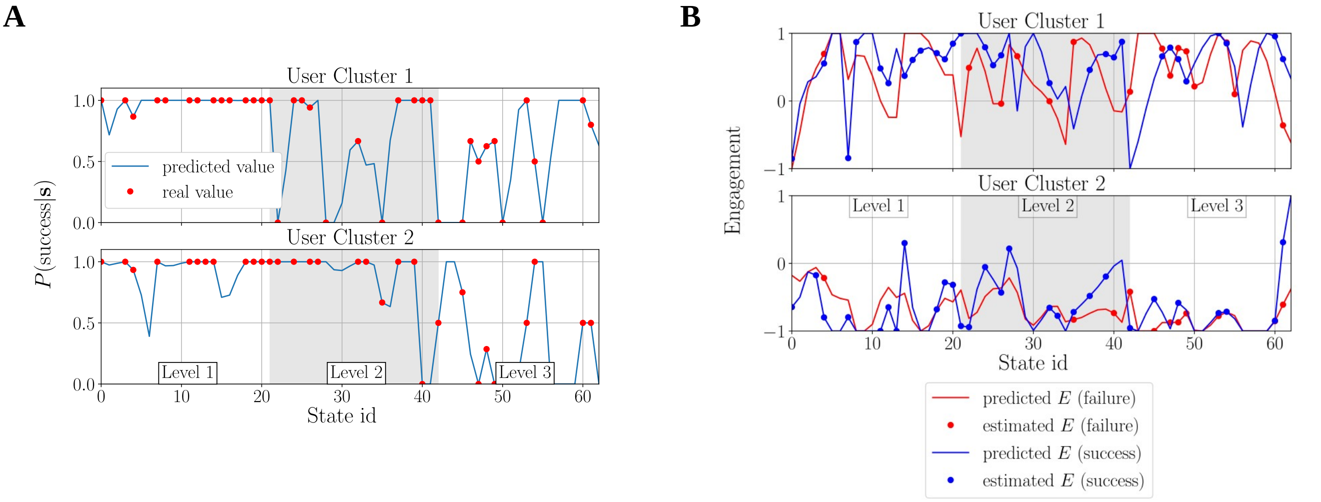

Figure S1. Regression problem solved with SVR for modelling **(A)** activity performance and **(B)** engagement

It is important to mention that we evaluated the trained models on the training data and chose the final one based on a visual observation, as there is a relatively little amount of collected data. This is especially the case for the engagement estimates for C_1 given the user made a mistake in the easiest sequence (the sequence of three words was easy to memorise and most of the participants solved it correctly). In addition, the values for the most difficult sequences (game level 3) (Fig. S1) **(A)** vary significantly and splitting them into training and test sets can negatively impact the model's ability to accurately represent the data.

2 BEHAVIOUR MODEL

2.1 Feedback Types

The form of encouraging and challenging feedback depends on the result of recreating the sequence by a user (success or failure). The performed gestures and facial expressions, as well as the said sentences by the robot for each case, are depicted in Table S3. Additionally, to make the robot's behaviour more

Table S3. Gestures, face expressions and sentences used for giving feedback to the user. The field *result* indicates if the user recreated correctly (success) or wrongly (failure) the sequence of emotions previously said by the robot.

**Result**	**Feedback Type**					
	**Encouraging**			**Challenging**		
	**Gesture**	**Face Expression**	**Sentences**	**Gesture**	**Face Expression**	**Sentences**
success	`hoorah`	`happy`	”That was great! Keep up the good work!”	`happy`	`one_eye_wink`	”OK, that was easy enough! Let's see now ...”
failure	`surprised`	`neutral`	”Oh, that was wrong! But that's fine! Don't give up!”	`calm`	`confused`	”Hey! Are you there? Stay focused when I speak!”

involving, we programmed it to give non-verbal feedback as well. We used four types of gestures provided by the QTrobot API: `hoorah`, `happy`, `calm`, `surprised`. The first gesture is the most complex; thus,

we depict its entire execution in Fig. S2. The poses depicted in Figure **(A)** and Figure **(E)** are common

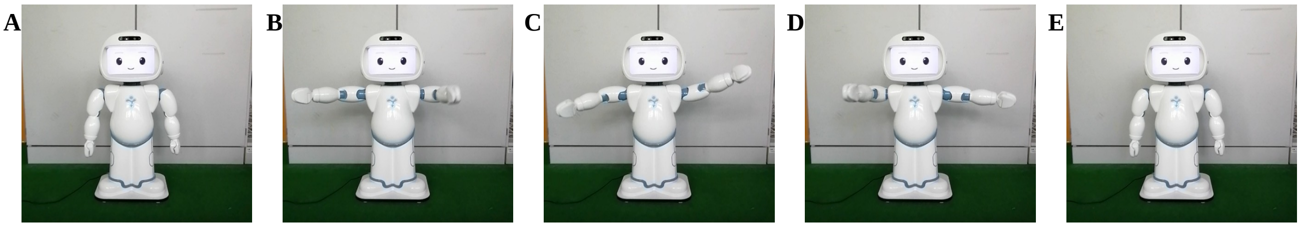

Figure S2. Visualization of the execution of the entire `hoorah` feedback gesture (read left-to-right). Figures **(A)** and **(E)** represent the starting and final robot poses accordingly, which are common for all types of feedback gestures. Figures **(B)**-**(D)** represent all the intermediate robot poses.

for all the gestures; thus, for other ones, we show only the intermediate robot poses in Fig. S3. We

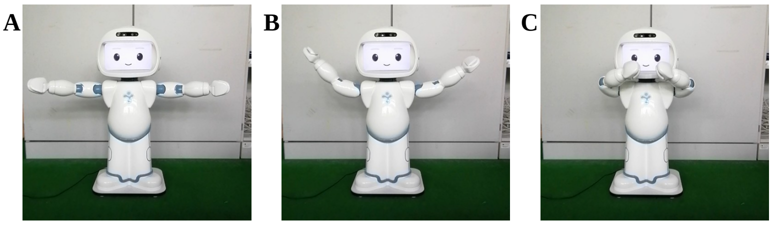

Figure S3. Robot positions for the **(A)** `calm`, **(B)** `happy` and **(C)** `surprised` feedback gestures

used four types of robot face expressions provided by the QTrobot API as well: `happy`, `neutral`, `one_eye_wink`, `confused`. All of them are depicted in Fig. S4. It should be mentioned that the default

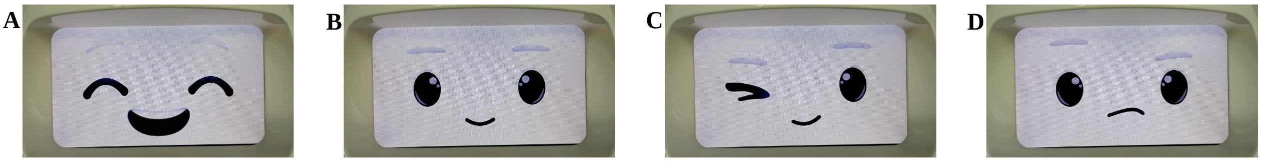

Figure S4. Robot face expressions (A) `happy`, (B) `neutral`, (C) `one_eye_wink` and (D) `confused`.

robot face expression is `neutral`, which means that the robot is displaying it, for example, while saying the sequence of emotions or waiting for the user to recreate the sequence. Using the neutral face essentially means that the robot did not get a command to show any other face expressions.

2.2 Obtained Policies

The behaviour model policies trained for both user clusters (C_1 and C_2) are depicted in Fig. S5, Fig. S6, Fig. S7, Fig. S8, Fig. S9, Fig. S10. Formulas S4 are repeated here (they were already given in the article) for clarity.

$$RE_{d,t} \leftarrow \begin{cases} 2L_t & \text{if } O_t = 1 \\ -1 & \text{if } O_t = -1 \end{cases}, RE_{s,t} \leftarrow \begin{cases} L_t^2 & \text{if } O_t = 1 \\ -1 & \text{if } O_t = -1 \end{cases} \tag{S4}$$

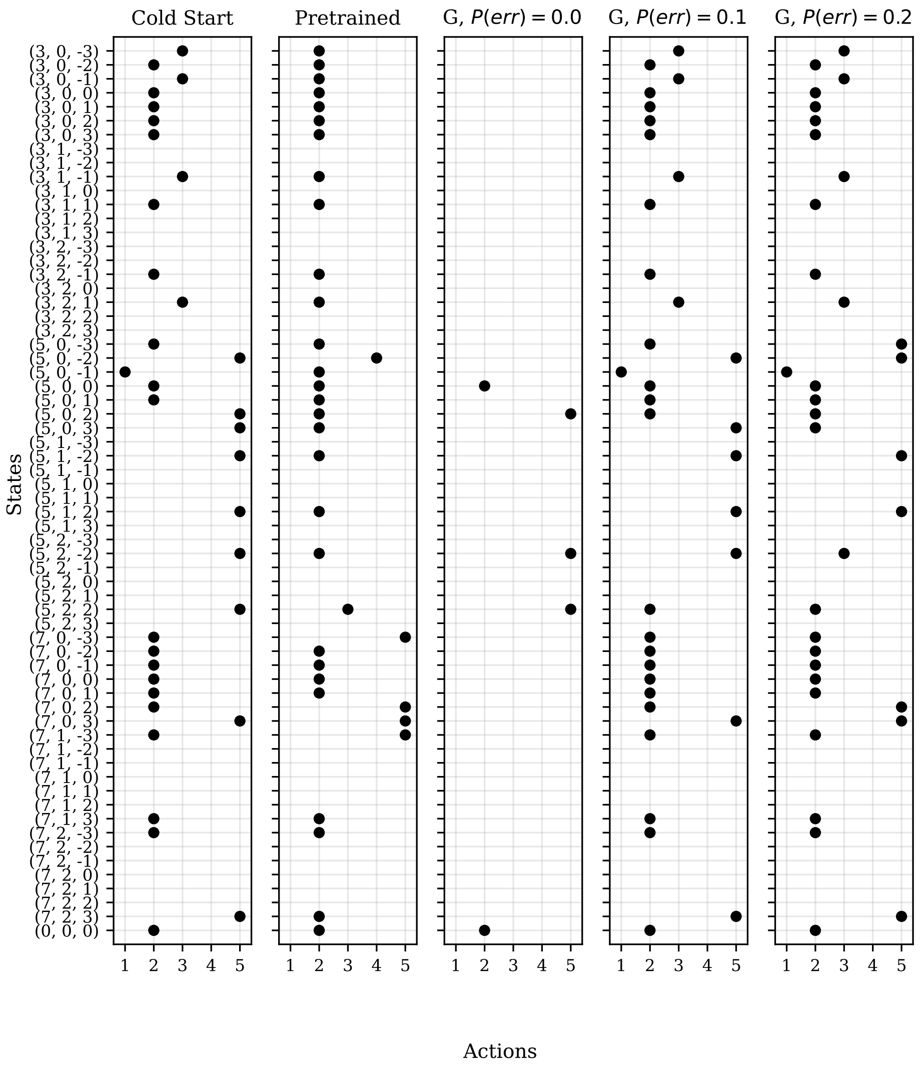

Figure S5. Policy visualization for C_1. The reward function is calculated with the original RE and $\beta = 3$. The actions 1-3 stand for applying a sequence of length of 3,5,7 respectively. The actions 4,5 stand for giving encouraging and challenging feedback respectively.

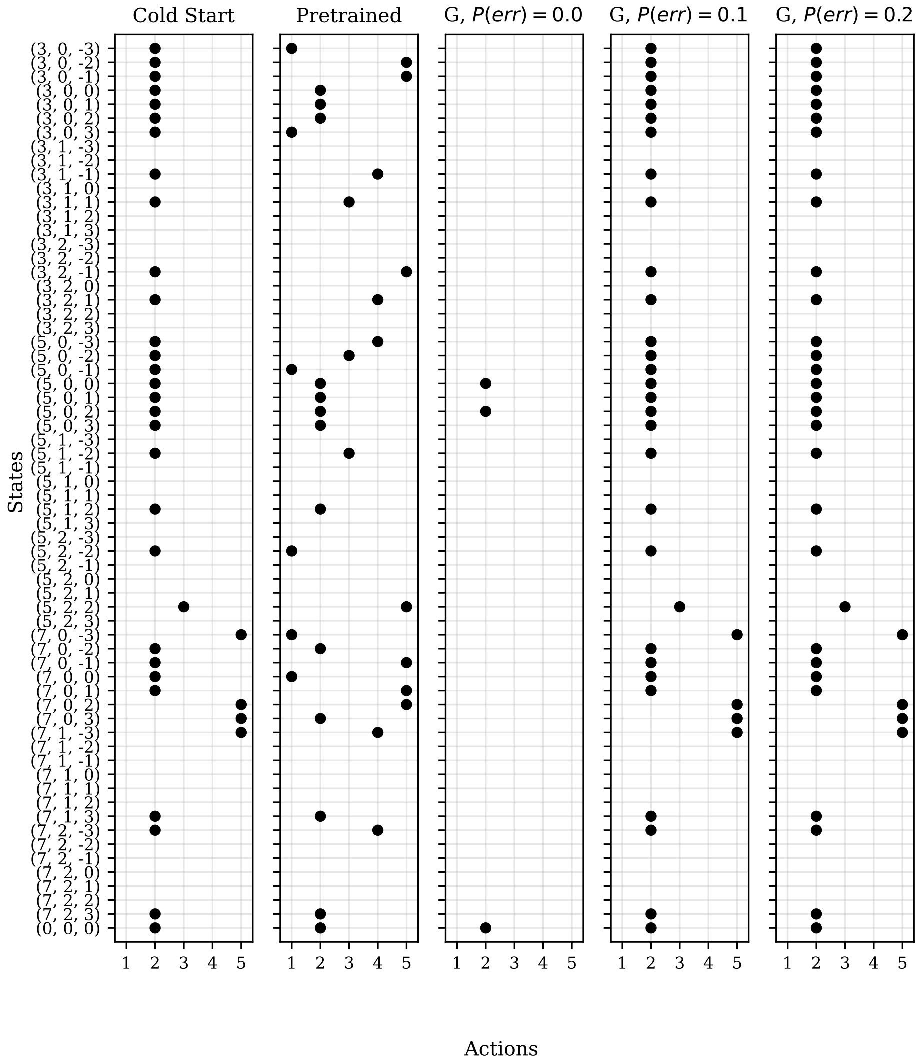

Figure S6. Policy visualization for C_2. The reward function is calculated with the original RE and $\beta = 3$. The actions 1-3 stand for applying a sequence of length of 3,5,7 respectively. The actions 4,5 stand for giving encouraging and challenging feedback respectively.

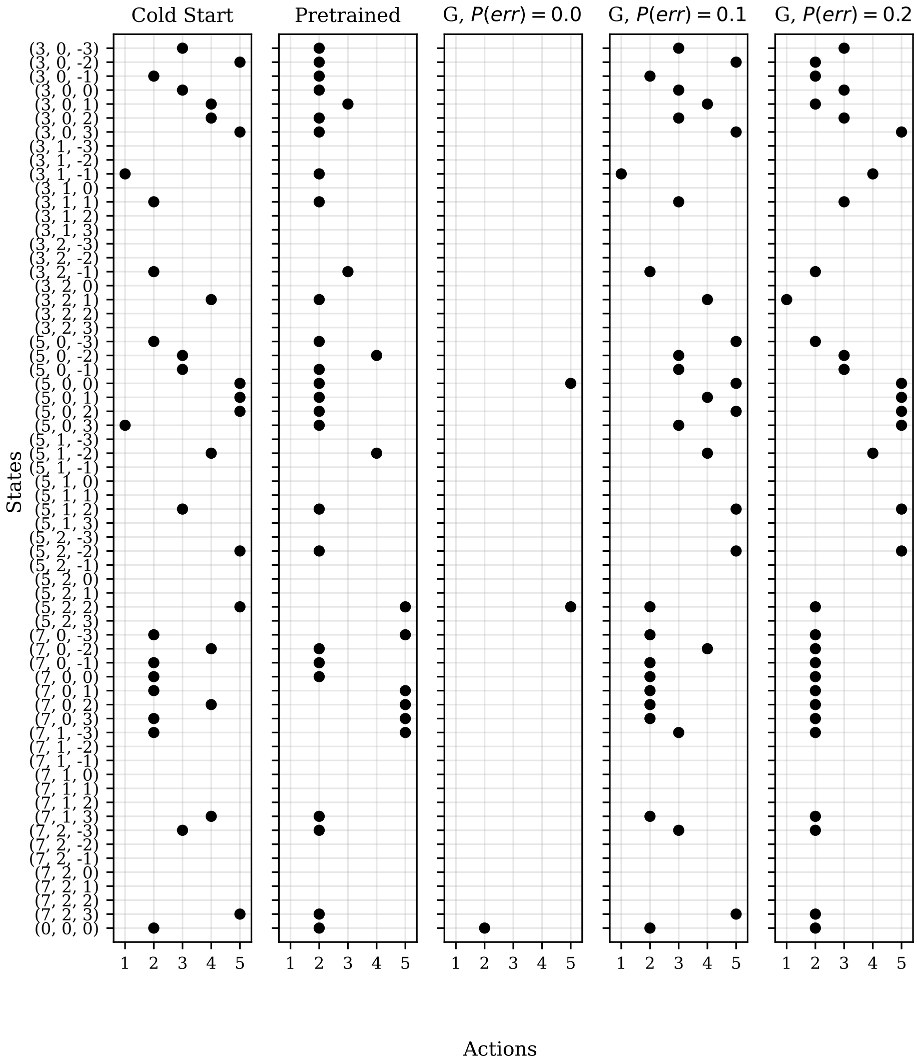

Figure S7. Policy visualization for C_1, with $RE_{d,t}$, $\beta = 5$ for both cold start and pretrained condition; and $\beta = 3$ for guidance. The actions 1-3 stand for applying a sequence of length of 3,5,7 respectively. The actions 4,5 stand for giving the encouraging and challenging feedback respectively.

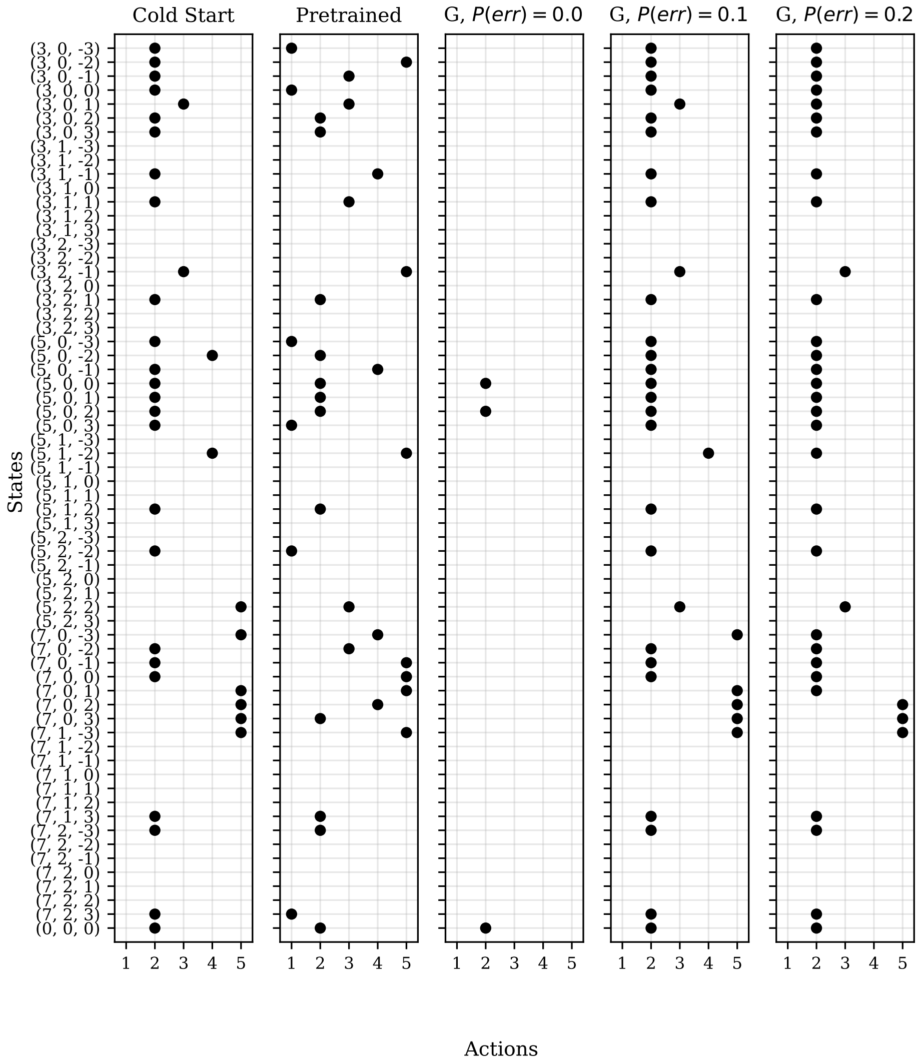

Figure S8. Policy visualization for C_2, with $RE_{d,t}$, $\beta = 5$ for both cold start and pretrained condition; and $\beta = 3$ for guidance. The actions 1-3 stand for applying a sequence of length of 3,5,7 respectively. The actions 4,5 stand for giving the encouraging and challenging feedback respectively.

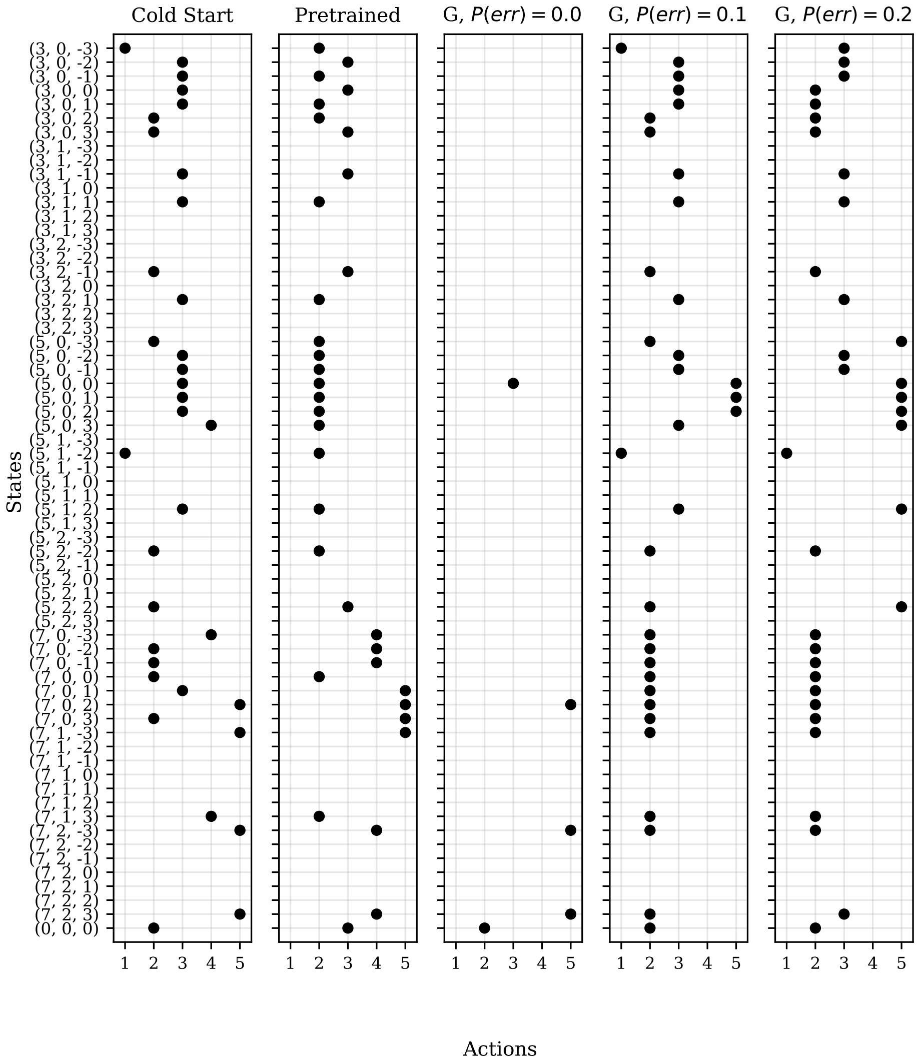

Figure S9. Policy visualization for C_1, with $RE_{s,t}$, $\beta = 8$ for both cold start and pretrained condition; and $\beta = 3$ for guidance. The actions 1-3 stand for applying a sequence of length of 3,5,7 respectively. The actions 4,5 stand for giving encouraging and challenging feedback respectively.

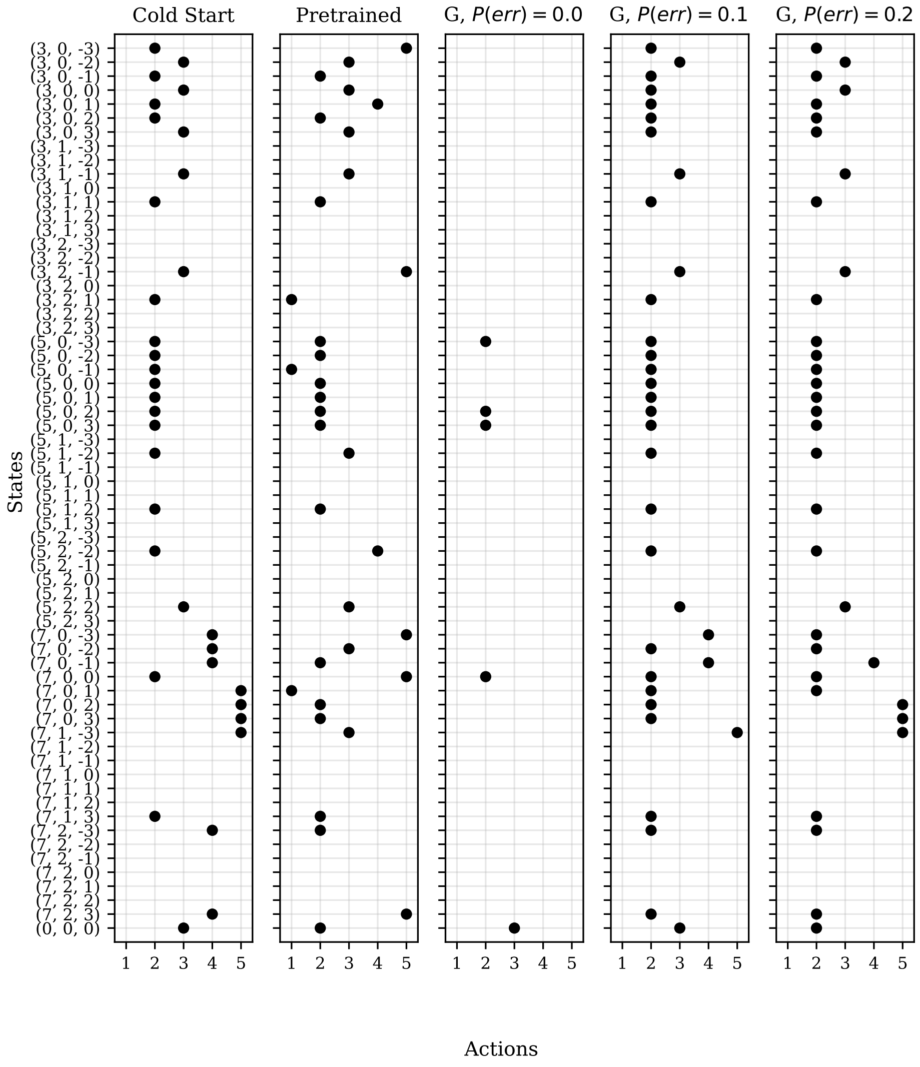

Figure S10. Policy visualization for C_2, with $RE_{s,t}$, $\beta = 8$ for both cold start and pretrained condition; and $\beta = 3$ for guidance. The actions 1-3 stand for applying a sequence of length of 3,5,7 respectively. The actions 4,5 stand for giving encouraging and challenging feedback respectively.

3 RESULTS

It is important to mention that some users did not behave as expected due to environmental disturbances or the way they were focusing on the robot; for instance, some users preferred to listen to the robot with closed eyes rather than look at it, which affects the engagement estimate. The expected engagement E during one session for all the participants (except the one for which E has already been presented in the article) is depicted in Fig. S11, Fig. S12, Fig. S13, Fig. S14, Fig. S15, Fig. S16, Fig. S17, Fig. S18, Fig. S19, Fig. S20, Fig. S21, Fig. S22, Fig. S23, Fig. S24, Fig. S25, Fig. S26, Fig. S27, Fig. S28, Fig. S29. . The black crosses are times when the user had to recreate a sequence on the tablet; the red lines mark the ends of game stages (with a certain L), which are followed by either the start of a new sequence or robot feedback given to the user.

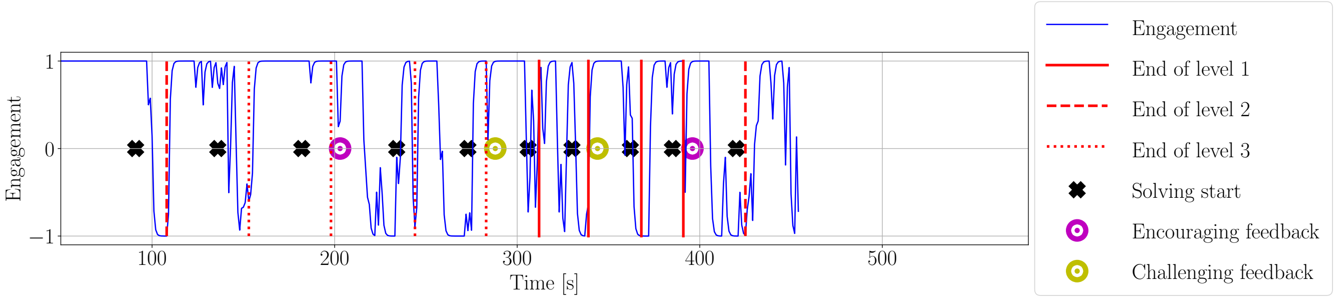

Figure S11. Expected E in one session for participant no 0.

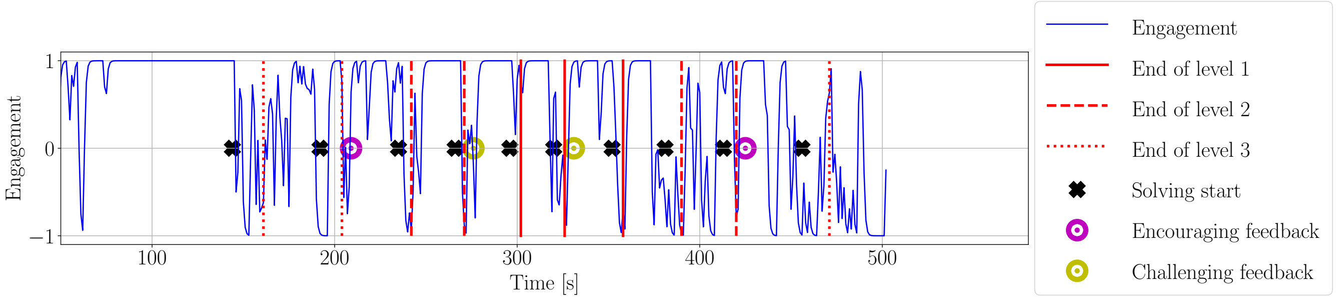

Figure S12. Expected E in one session for participant no 1.

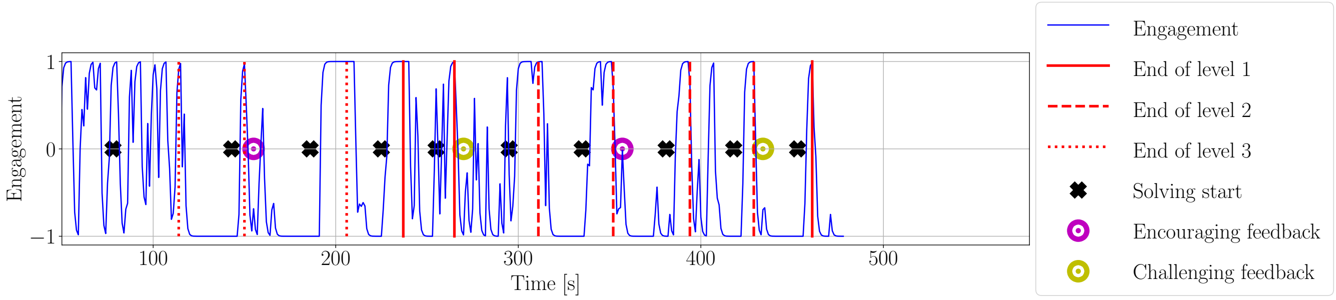

Figure S13. Expected E in one session for participant no 2.

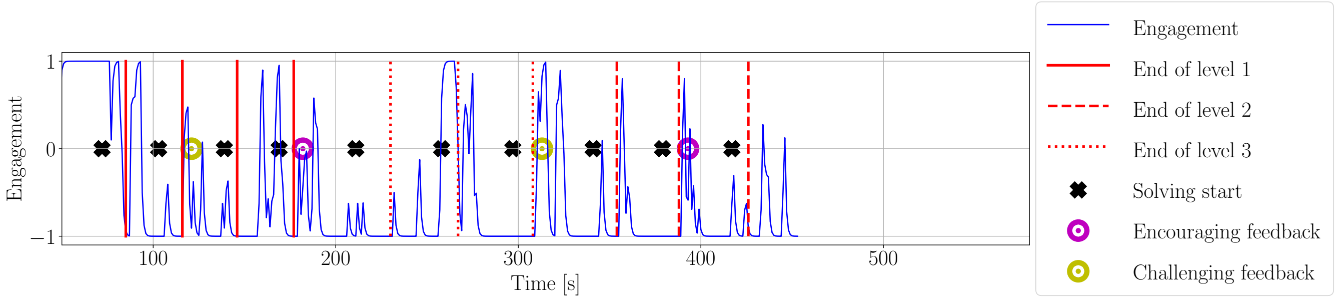

Figure S14. Expected E in one session for participant no 3.

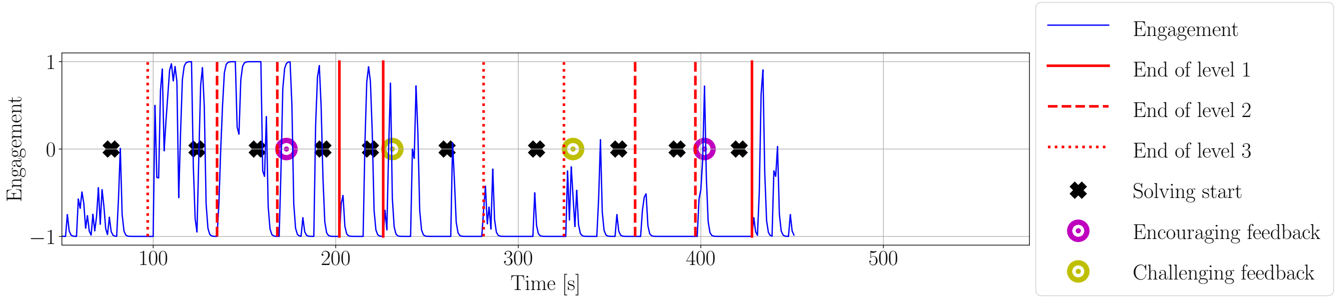

Figure S15. Expected E in one session for participant no 4.

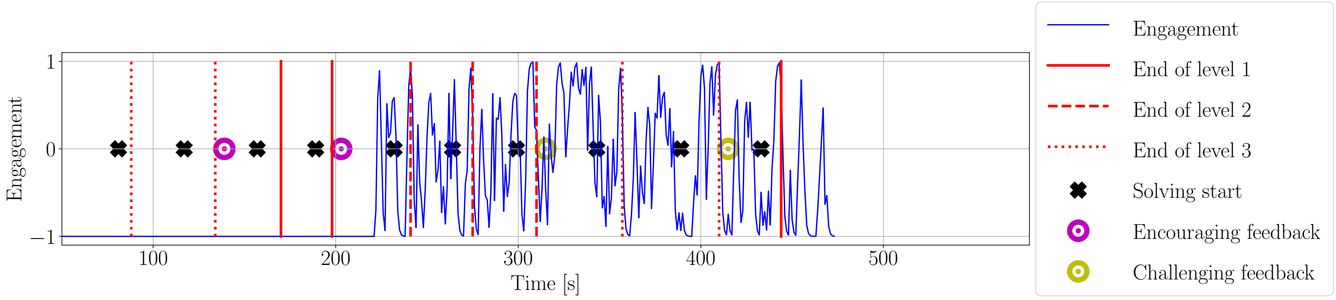

Figure S16. Expected E in one session for participant no 5.

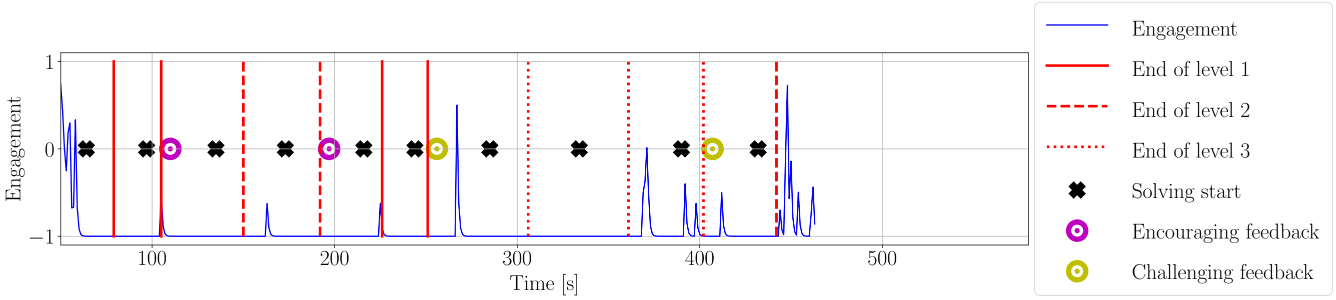

Figure S17. Expected E in one session for participant no 6.

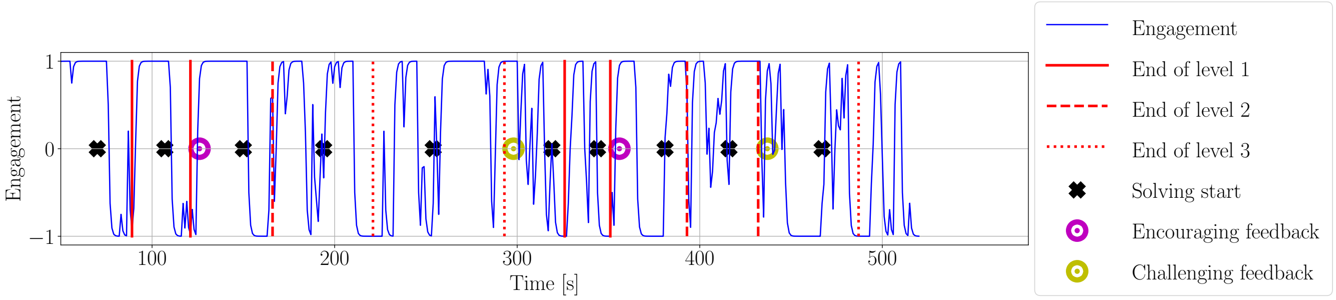

Figure S18. Expected E in one session for participant no 7.

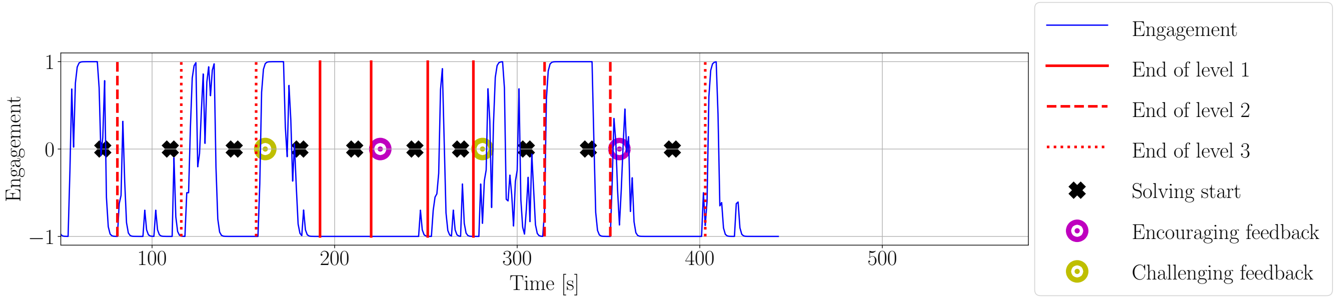

Figure S19. Expected E in one session for participant no 8.

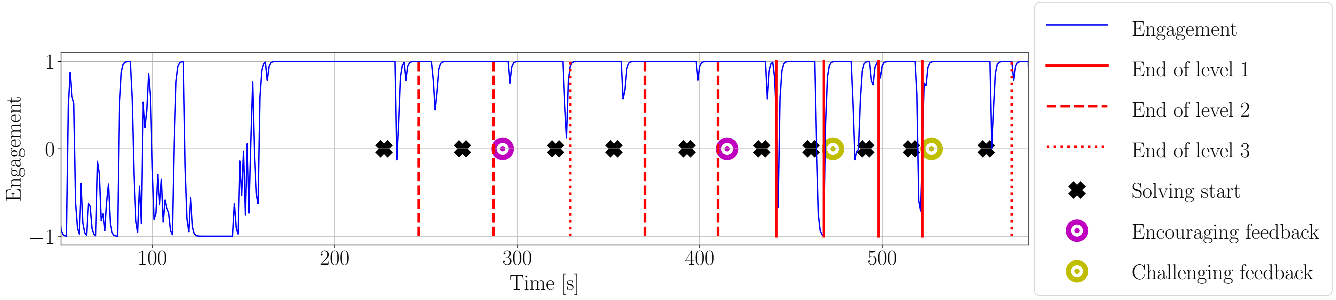

Figure S20. Expected E in one session for participant no 9.

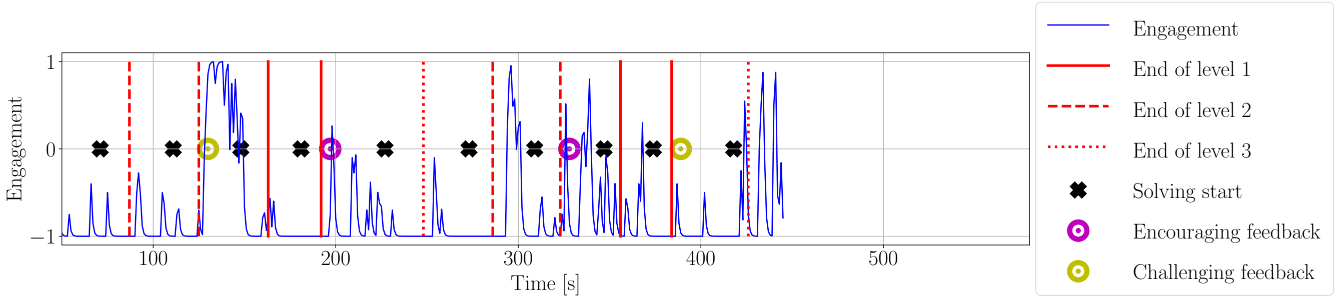

Figure S21. Expected E in one session for participant no 10.

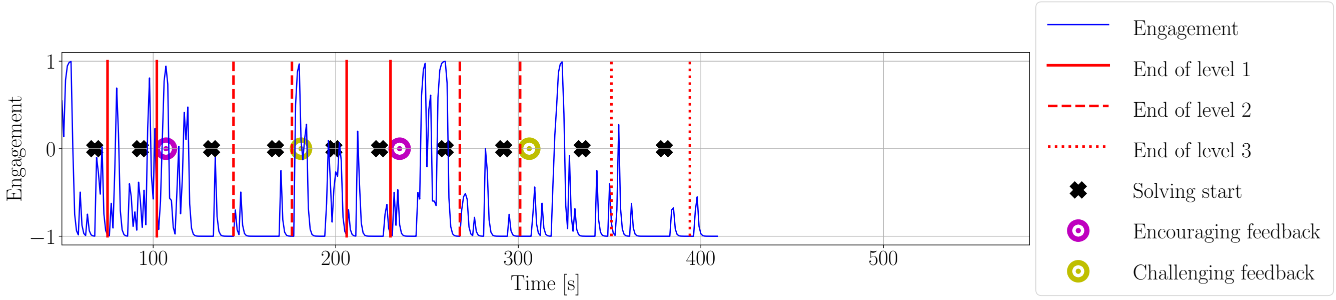

Figure S22. Expected E in one session for participant no 11.

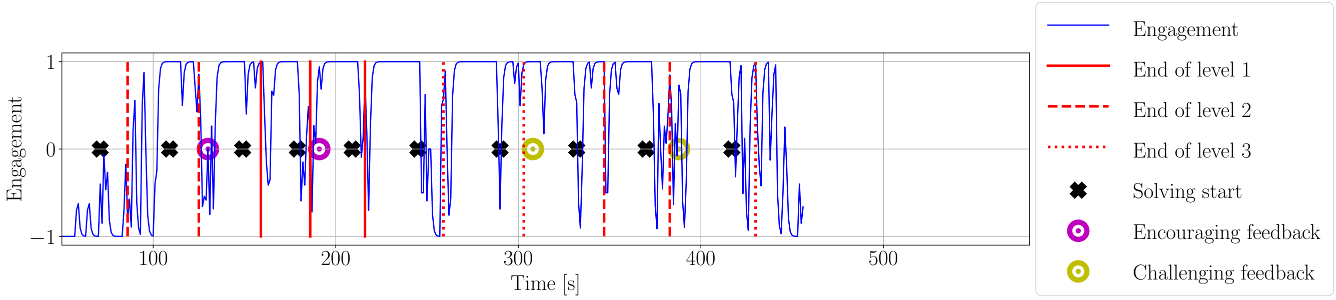

Figure S23. Expected E in one session for participant no 13.

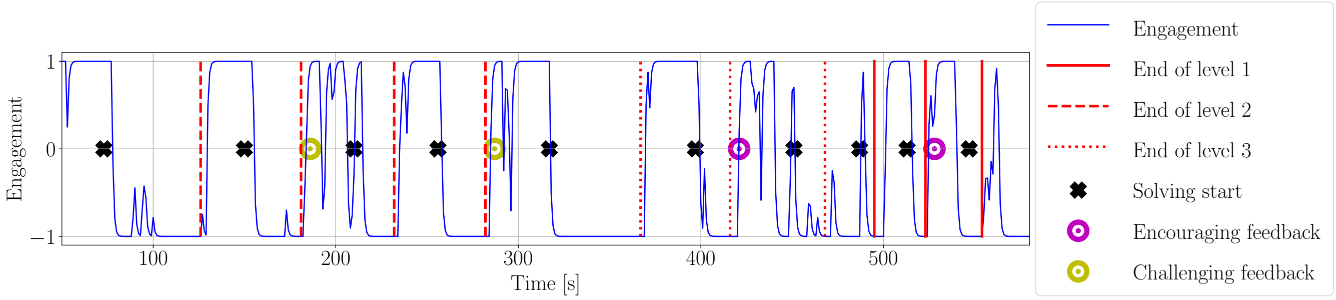

Figure S24. Expected E in one session for participant no 14.

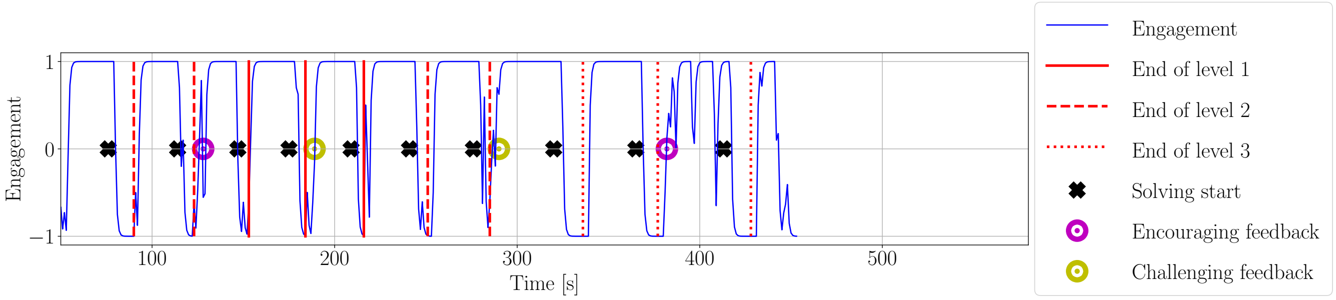

Figure S25. Expected E in one session for participant no 15.

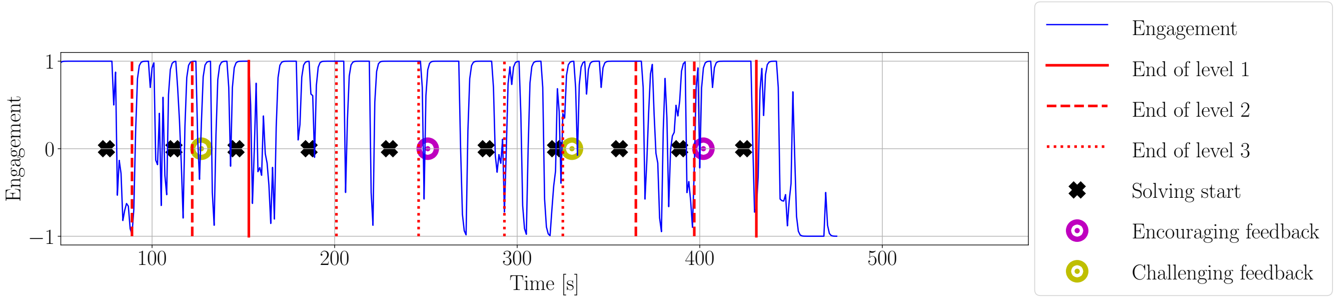

Figure S26. Expected E in one session for participant no 16.

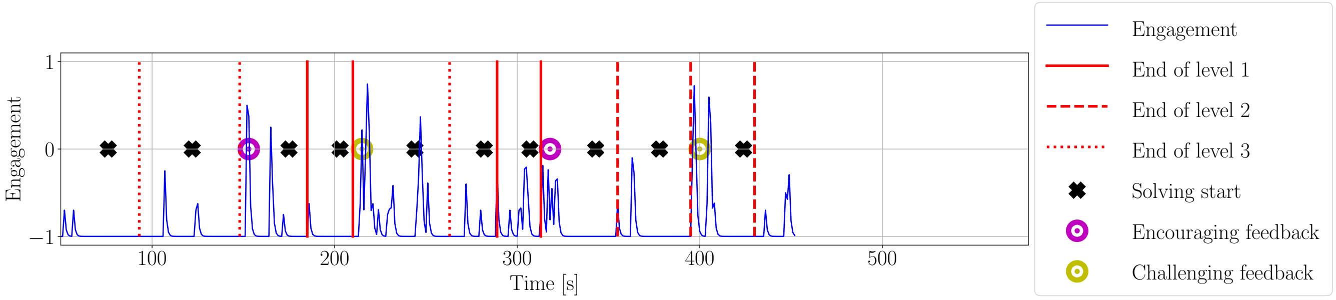

Figure S27. Expected E in one session for participant no 17.

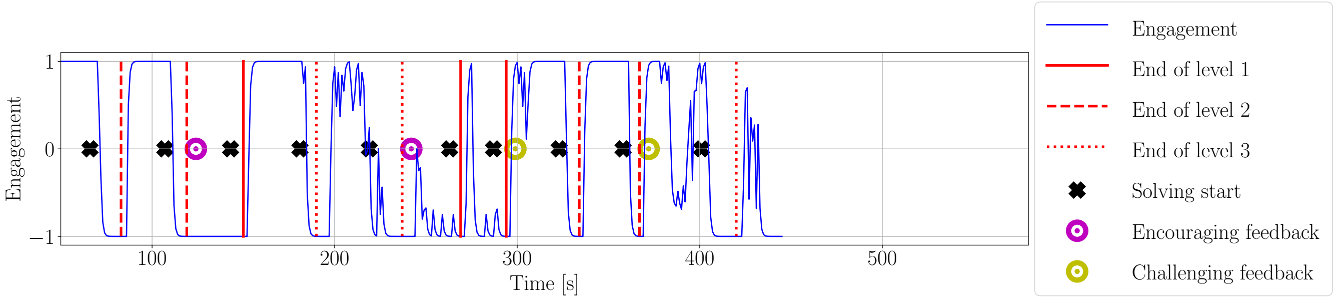

Figure S28. Expected E in one session for participant no 18.

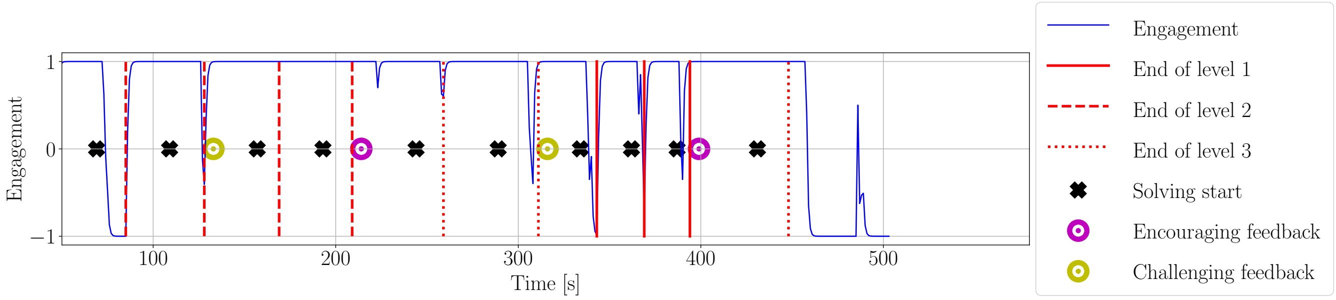

Figure S29. Expected E in one session for participant no 19.
